# Improving Primary Health Care in Chronic Musculoskeletal Conditions through Digital Media: The PEOPLE Meeting

**DOI:** 10.2196/resprot.2267

**Published:** 2013-03-08

**Authors:** Linda C Li, Cheryl Cott, C Allyson Jones, Elizabeth M Badley, Aileen M Davis, PEOPLE Meeting Participants

**Affiliations:** ^1^Arthritis Research Centre of CanadaRichmond, BCCanada; ^2^Department of Physical TherapyUniversity of British ColumbiaVancouver, BCCanada; ^3^Toronto Western Research InstituteToronto, ONCanada; ^4^Department of Physical TherapyUniversity of AlbertaEdmonton, ABCanada; ^5^see acknowledgements for contributors

**Keywords:** primary health care, Internet, digital media, health service delivery

## Abstract

**Background:**

Musculoskeletal (MSK) conditions are the most common cause of severe chronic pain and disability worldwide. Despite the impact of these conditions, disparity exists in accessing high quality basic care. As a result, effective treatments do not always reach people who need services. The situation is further hampered by the current models of care that target resources to a limited area of health services (eg, joint replacement surgery), rather than the entire continuum of MSK health, which includes services provided by primary care physicians and health professionals. The use of digital media offers promising solutions to improve access to services. However, our knowledge in this field is limited. To advance the use of digital media in improving MSK care, we held a research planning meeting entitled “PEOPLE: *P*artnership to *E*nable *O*ptimal *P*rimary Health Care by *Le*veraging Digital Media in Musculoskeletal Health”. This paper reports the discussion during the meeting.

**Objective:**

The objective of this study was to: (1) identify research priorities relevant to using digital media in primary health care for enhancing MSK health, and (2) develop research collaboration among researchers, clinicians, and patient/consumer communities.

**Methods:**

The PEOPLE meeting included 26 participants from health research, computer science/digital media, clinical communities, and patient/consumer groups. Based on consultations with each participant prior to the meeting, we chose to focus on 3 topics: (1) gaps and issues in primary health care for MSK health, (2) current application of digital media in health care, and (3) challenges to using digital media to improve MSK health in underserviced populations.

**Results:**

The 2-day discussion led to emergence of 1 overarching question and 4 research priorities. A main research priority was to understand the characteristics of those who are not able to access preventive measures and treatment for early MSK diseases. Participants indicated that this information is necessary for tailoring digital media interventions. Other priorities included: (1) studying barriers and ethical issues associated with the use of digital media to optimize MSK health and self-management, (2) improving the design of digital media tools for providing “just-in-time” health information to patients and health professionals, and (3) advancing knowledge on the effectiveness of new and existing digital media interventions.

**Conclusions:**

We anticipate that the results of this meeting will be a catalyst for future research projects and new cross-sector research partnerships. Our next step will be to seek feedback on the research priorities from our collaborators and other potential partners in primary health care.

## Introduction

Musculoskeletal (MSK) conditions are the most common cause of severe chronic pain and disability worldwide [1-4]. Approximately 33% of adults in the United States reported having chronic joint symptoms and arthritis [5]. In Canada, arthritis alone affects 4.4 million people (age ≥ 15 years) and is projected to affect 7 million in 20 years [2]. Effective treatments are available. For example, strong evidence indicates that lifestyle interventions such as exercise [6-8], weight management [9-11], and education [12,13] can reduce pain, improve quality of life [6,14,15], and have potential to reduce the progression of joint damage in those with osteoarthritis [16]. Also, there is ample evidence supporting the early and consistent use of disease modifying medications for treating inflammatory arthritis [17-19]. However, despite the evidence, most people in Canada do not receive the basic care in a timely manner to maintain MSK health [20-22] or cannot access treatment at all [23-25]. The situation is further hampered by our current models of care that invest in a limited aspect of services provided by specialists (eg, triaging for early diagnosis of inflammatory arthritis or provision of joint replacement surgery), rather than the entire continuum of MSK health [26], which includes treatments and health promotion strategies provided by a variety of primary care health professionals [27].

The use of digital media offers promising solutions to improve access to services. Digital media, which are electronic media that operate on digital codes, offer a broad range of applications, such as social networking tools, online games, animation, interactive websites, and mobile applications. They provide tremendous flexibility for delivering health-related information at a time and place that is chosen by the individual. In the past, digital media referred to “new media” for specific applications. Today, digital media is everywhere. In contrast, our knowledge on their use in health promotion, treatment, and self-management is still in its infancy. To advance the use of digital media in improving MSK care, we held a research planning meeting on October 13-14, 2011 in Vancouver, Canada, entitled “PEOPLE: *P*artnership to *E*nable *O*ptimal *P*rimary Health Care by *Le*veraging Digital Media in Musculoskeletal Health”. The purpose of this paper was to report the research priorities generated from this meeting.

## Pre-meeting Consultation

The PEOPLE meeting was supported by the Canadian Institutes of Health Research (CIHR)-funded Model of Care in Arthritis Team (CIHR Funding Reference Number: EMT-92253) [28]. Twenty-six invitees, representing health and computer science disciplines, clinical communities, and patients/consumers groups participated at this meeting. All participants were considered equal contributors in discussions and the development of research priorities. Before the meeting, a series of individual consultations were held by the co-chairs (Linda Li and Aileen Davis) to learn about participants’ expectations of the meeting, and their opinions regarding the use of digital media in primary health care. Comments from each participant were reviewed to develop new probing questions for subsequent consultations.

Overall, participants agreed that the meeting should focus on three areas: (1) gaps and issues in primary care for maintaining/improving MSK health, (2) successes and pitfalls of current applications of digital media in health care, and (3) challenges to using digital media for improving primary health care in underserviced populations.

## PEOPLE Meeting

The PEOPLE meeting consisted of 3 sessions; each included short, thought-provoking presentations and facilitated discussions (Multimedia Appendix 1). The meeting ended with a group brainstorming on research priorities. At the start, participants agreed to adopt Health Canada’s definitions of “primary care” and “primary health care”. Primary care was defined as the point of first contact with the health care system. It includes “the diagnosis, treatment, and management of health problems with services delivered predominantly by physicians, but increasingly by other professionals with direct patient access” [29].

Health Canada defined primary health care as “an approach to providing health care that involves health professionals working together and delivering care within the context of the broader determinants of health of individuals and communities”. As such, primary health care encompasses a range of services, “including illness prevention, health promotion, diagnosis, and management of health concerns. It encourages the use of the health professional(s) from the most appropriate health discipline(s) to maximize the potential of health resources” [30].

## Session 1: Gaps and Issues in Primary Health Care for Musculoskeletal Health

The session began with a presentation on the current practice, challenges, and opportunities in MSK care (by Dr. Thea Vliet Vlieland). This talk highlighted recent research that used Web-based interventions to improve arthritis care in The Netherlands. This was followed by Ms. Simone Hughes, who shared her past experience as a person with arthritis living in a rural community, and Ms. Robin Roots, who presented her research on rural and remote rehabilitation practice. The final presentation discussed the use of digital media from a physician’s perspective (by Dr. Preston Wiley). Participants were asked to reflect on 2 questions after each presentation: 1) What are the issues in primary health care that can/should be addressed by research? 2) How may digital media be applied to improve primary health care?

An issue raised during the discussion was the difficulties experienced by individuals, their families, and health professionals in accessing high quality information about the services required or available to manage MSK health. It was suggested that in areas where there were physician shortages, nurse practitioners and advanced practice rehabilitation professionals might help to provide care and assist patients to access services. Participants also pointed out that although well-designed digital media tools could help to bridge the information gap, underserviced populations might be the least likely to know about, and have access to, computers and/or mobile devices for information. To quote one participant, “If you build it, they may not come.” Therefore, a survey on how people who are currently underserviced access information would be useful.

The group also discussed challenges posed by existing computerized tools for accessing health information (ie, design issues). Participants with a computer science/design background noted that most tools in the health sector were not user friendly, and some were developed without target audiences in mind. They believed that the future digital media tools should be designed to provide customized information, taking into account users’ needs and literacy levels. The group also raised concerns about accessing information using digital media portals, particularly with the growing “broadband divide” between those living in the city and those living in areas with unreliable broadband Internet access. This issue needs to be taken into consideration when designing tools to support primary health care and patient self-management in rural and remote areas.

## Session 2: Current Application of Digital Media in Health Care

This session began with an overview on research that uses virtual reality, robotics, and social networking programs to manage chronic pain and anxiety disorders (by Drs. Diane Gromala and Chris Shaw). Participants also heard about the research on communication technology and health (Dr. Sherida Ryan). Dr. Ryan raised an important issue of the poor “cyber literacy” of the general population, which included not knowing how to determine the credibility of a website or how to protect one’s personal information and identity on the Internet. Finally, Dr. Scott Lear shared his research on telehealth in chronic disease self-management in Northern British Columbia. Dr. Scott stressed the importance of attending to the technical issues and creating a navigation pathway for the online intervention at the start of a project.

After each presentation, participants were asked to reflect on the following question: How might digital media address issues identified in primary health care? Participants gave a few examples to illustrate how digital media could be used, including: (1) health surveillance (eg, provide monitoring and feedback to patients’ health status and lifestyle behaviors, such as participation in physical activity), (2) patient-health professional communication (eg, emails, Skype), (3) record keeping and health professional communication (eg, electronic medical record), (4) access knowledge (eg, patient education websites), and (5) continuing professional development (eg, e-communities of practice). In general, participants felt that further effort would be required to assist the public in judging the credibility of online health information. During the discussion about health surveillance, the concern of “self-management overload” and “monitor burden” was voiced by several patients/consumers. There was a tension between the need for monitoring and the focus on symptoms/illness. The latter might reinforce the sickness role and could have a detrimental effect on the individual.

It was suggested that future research could explore how to use digital media tools to provide “staged information” to enhance MSK health (ie, just enough information relevant to the specific stage of the disease, rather than “too much too early”). Also suggested was the need to study the effectiveness of digital media tools for providing personalized monitoring and feedback for patients with MSK disease.

## Session 3: Roles and Challenges of Using Digital Media for Improving Primary Health Care in Underserviced Populations

This session began with presentations from 3 different viewpoints on using digital media in the health sector. First, a health educator/a person living with arthritis shared her experience as a member of a primary health care team for homeless people and her view on using online and mobile tools for providing care (Ms. Louise Crane). Next, a computer scientist, Ms. Meehae Song, presented an overview of new digital media technologies for the health sector, especially for marginalized populations (eg, people living in poverty, sex workers). Examples included virtual reality training for chronic pain management, telemedicine, eHealth monitoring bracelets, mobile devices, and smartphone add-ons. Designers of these tools paid attention to optimizing user friendliness while minimizing costs. Finally, a medical sociologist discussed the ethical issues associated with access to technology for self-management (Dr. Anne Townsend). Central to Dr. Townsend’s talk was the tension between educating people to self-manage and supporting self-management. The former was based on a health professionals’ assumption that individuals lack the knowledge and skills to self-manage, and hence, need to be taught; while the latter took a patient’s perspective of needing access to resources and support to manage one’s health. Dr. Townsend discussed the potential ethical dilemma these different views might have on the design and implementation of digital media tools in primary health care.

Participants were asked to reflect on the following questions after the presentations: (1) What are the additional issues that need to be addressed to improve primary health care in the underserviced population? (2) What are the potential applications of digital media to improve primary health care in underserviced populations? (3) What ethical issues, which underline the use of digital media for primary health care in underserviced populations, need to be factored into future research?

A variety of topics were discussed in this session. The group began by cautioning against a narrow definition of underserviced population (eg, only focusing on those living in rural/remote areas). People who are underserviced tended to have similar characteristics. Although some demographic characteristics were well known (eg, geography, income, and education), others such as sex/gender role, race, and access to social capital and network had not been fully explored. A better understanding of these characteristics would help further advance research in digital media and primary health care. It was agreed upon that community-based research principles should be followed when working with underserviced populations. Further, future research should take into account the lived experienced of the target populations.

Participants also discussed the current model of self-management, which was a “deficit model” [31]. This model implied that people became ill because they did something wrong, and so interventions should focus on helping them to “do the right thing”. The assumption was that health professionals should help people “evolve” from a passive recipient of care to an active participant (ie, educating people how to self-manage). Yet in reality, people with chronic disease find ways to manage their conditions every day [32], and so interventions should be developed to support self-management instead. Participants felt that this was where digital media might play a role. Finally, to fully leverage the potential of new technologies, the group emphasized collaborations between the health and digital media sectors in future research.

## Discussion

During the brainstorming for research priorities, an overarching question emerged: *In the pursuit to optimize MSK health, do digital media offer viable solutions to reach those who are hard to reach?* The PEOPLE meeting participants recognized that a basic challenge to providing primary health care for MSK health was the lack of access to “just-in-time” knowledge. This refers to concise knowledge that is available when and where it is needed by an individual making a treatment, clinical or policy decision, or for maintaining good health. Several research priorities were identified, including:

1. Understanding the characteristics of individuals who are underserviced.
2. Studying barriers and ethical issues associated with the use of digital media tools by underserviced populations to optimize MSK health and support self-management.
3. Identifying design issues for developing new tools/modifying existing technologies to provide “just-in-time” information for users, with attention paid to addressing health literacy and cyber literacy.
4. Increasing knowledge on the effectiveness of new and existing digital media interventions, as well as different implementation strategies.

In conclusion, the PEOPLE meeting has identified research priorities for improving primary health care in chronic MSK conditions through digital media, with the input of a variety of stakeholders. Given that the late adopters have accounted for most of the new Internet users since the late 2000s [33], digital media is not only the future, but is already present. The collaboration between health and digital media sectors is crucial to modernize primary health care for the 21^st^ century. We anticipate that the findings from this meeting will be a catalyst for future projects and new cross-sector research partnerships, and welcome feedback on this report.

## Multimedia Appendix 1

PEOPLE meeting background.

## Abbreviations

CIHR: Canadian Institutes of Health Research
MSK: musculoskeletal
PEOPLE: Partnership to Enable Optimal Primary Health Care by Leveraging Digital Media in Musculoskeletal Health
