# Supplementary material for: Improving Primary Health Care in Chronic Musculoskeletal Conditions through Digital Media: The PEOPLE Meeting
Source: JMIR Res Protoc. 2013 Mar 8;2(1):e13. doi: 10.2196/resprot.2267 (PMC3628154; doi:10.2196/resprot.2267)
Supplement: Supplementary file 1 [file resprot_v2i1e13_app1.pptx]

## Slide 1
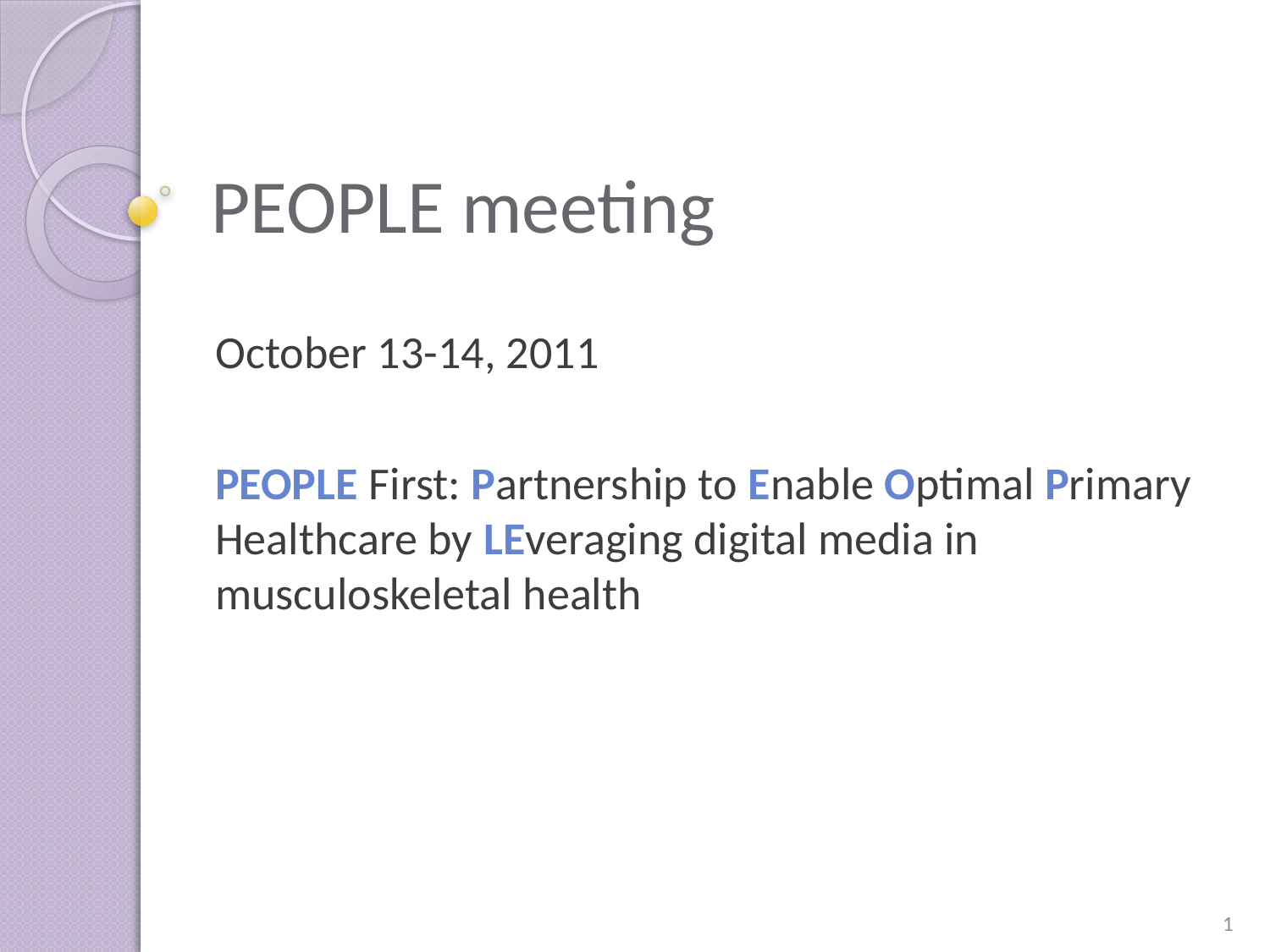

# PEOPLE meeting
October 13-14, 2011
PEOPLE First: Partnership to Enable Optimal Primary Healthcare by LEveraging digital media in musculoskeletal health
1

## Slide 2
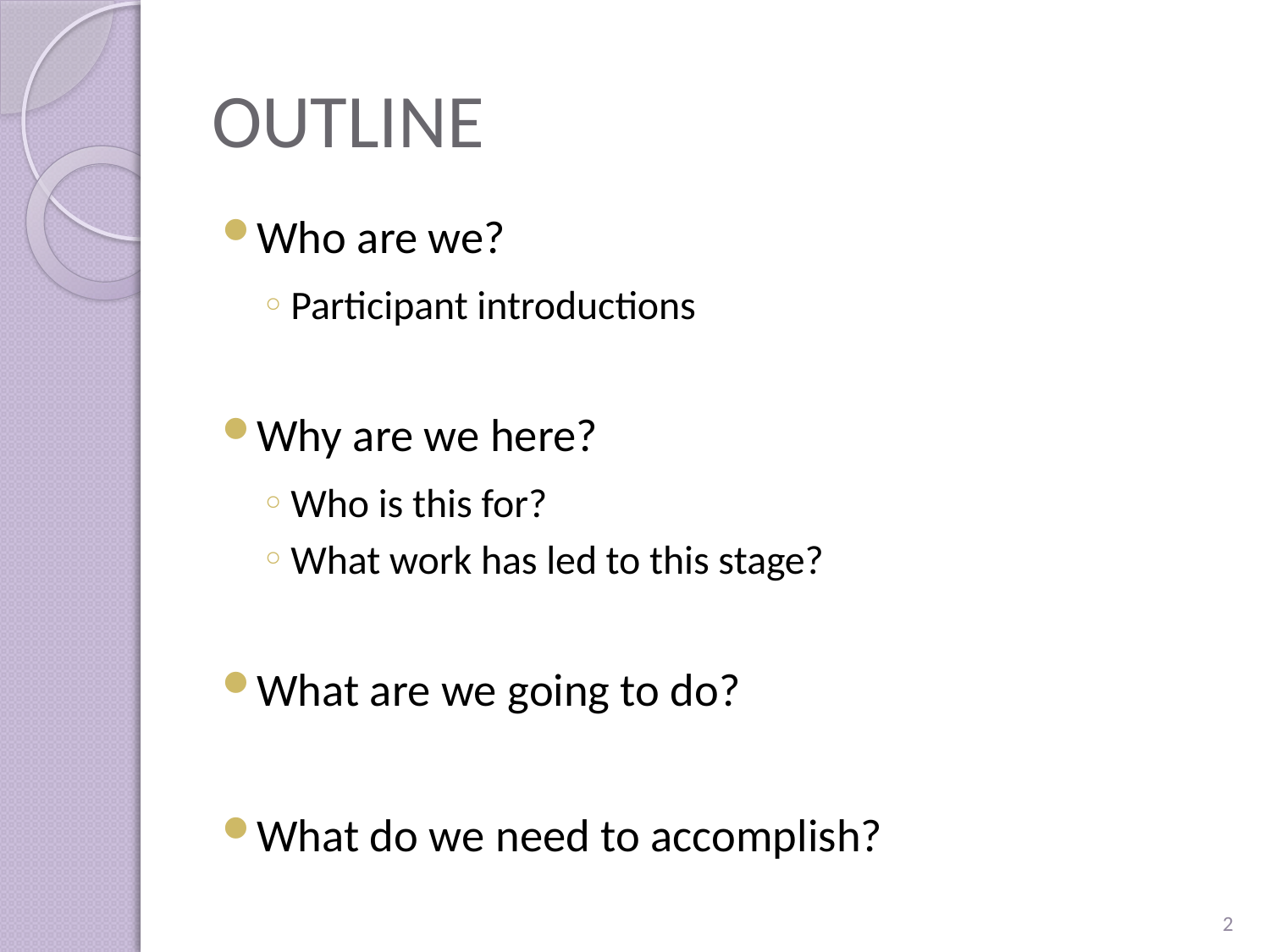

# OUTLINE
Who are we?
Participant introductions
Why are we here?
Who is this for?
What work has led to this stage?
What are we going to do?
What do we need to accomplish?
2

## Slide 3
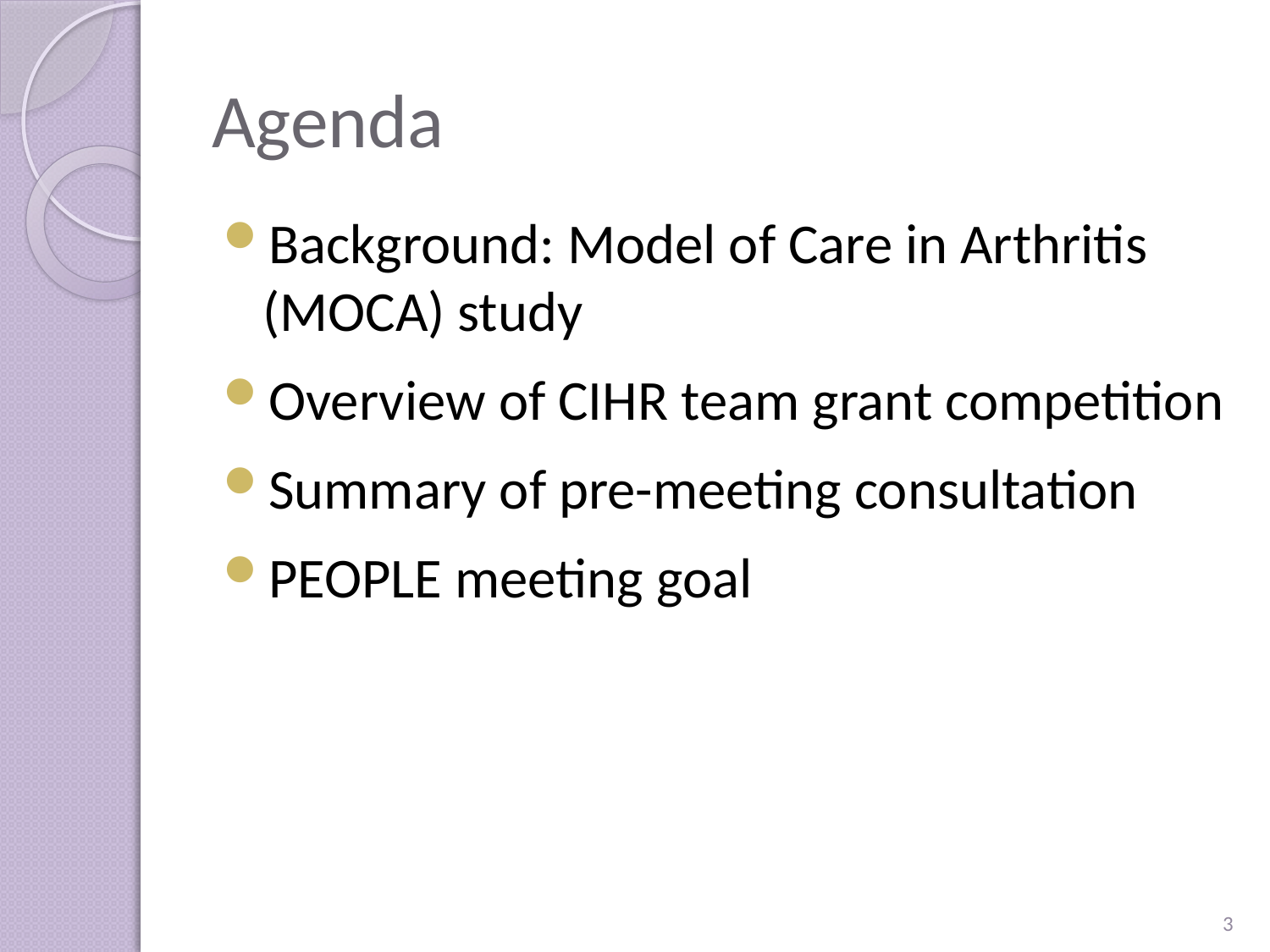

# Agenda
Background: Model of Care in Arthritis (MOCA) study
Overview of CIHR team grant competition
Summary of pre-meeting consultation
PEOPLE meeting goal
3

## Slide 4
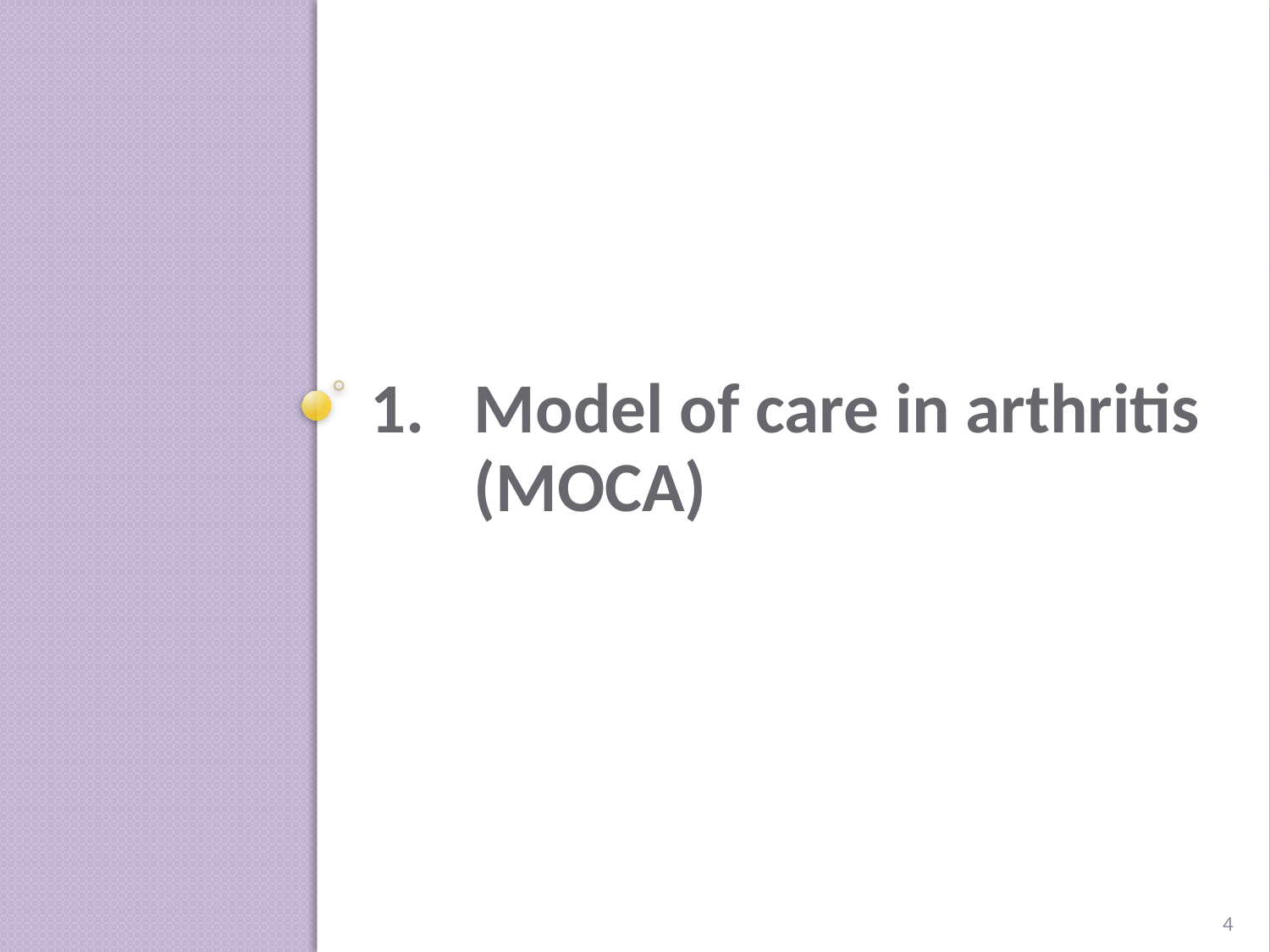

# Model of care in arthritis (MOCA)
4

## Slide 5
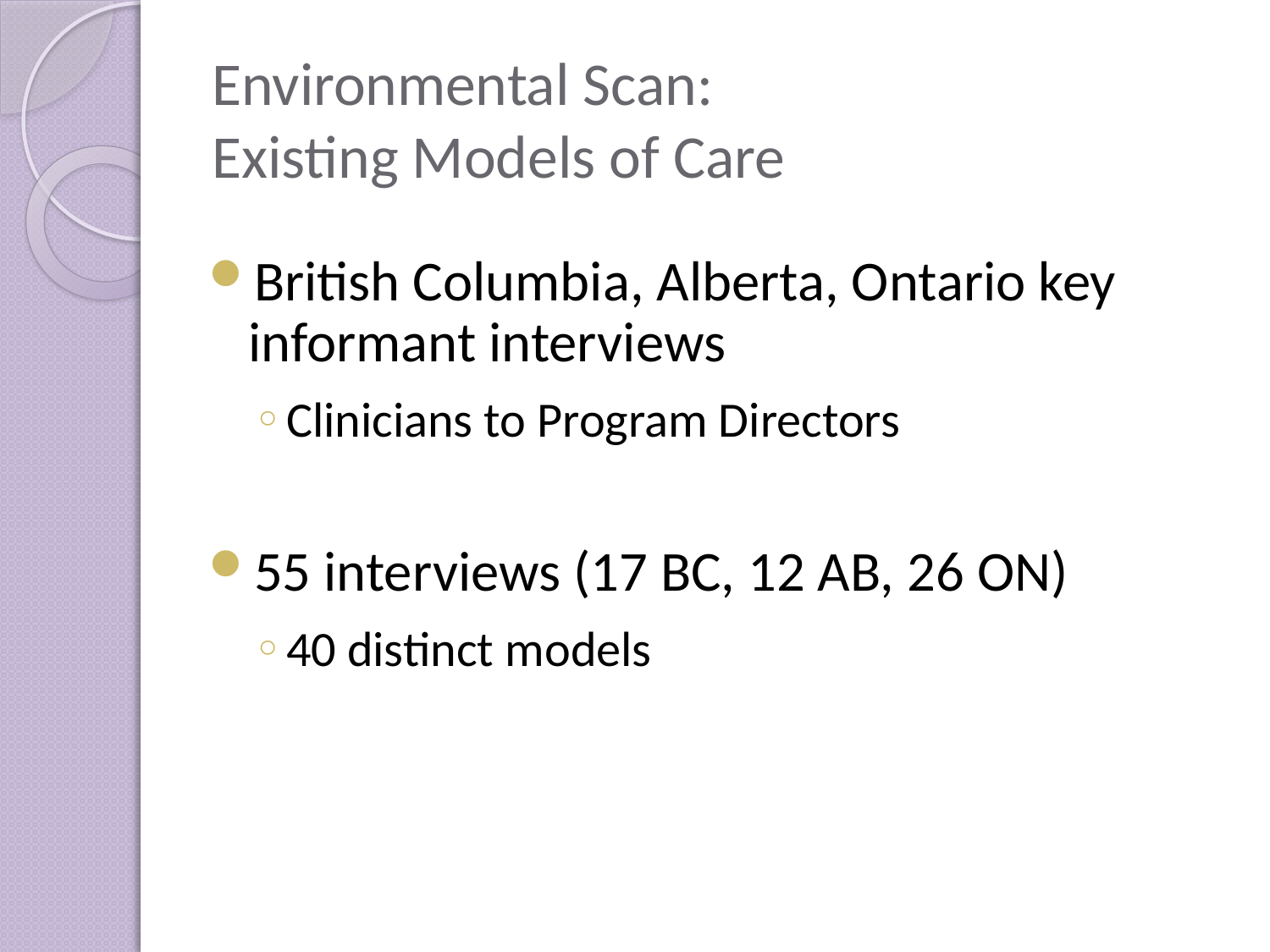

# Environmental Scan: Existing Models of Care
British Columbia, Alberta, Ontario key informant interviews
Clinicians to Program Directors
55 interviews (17 BC, 12 AB, 26 ON)
40 distinct models

## Slide 6
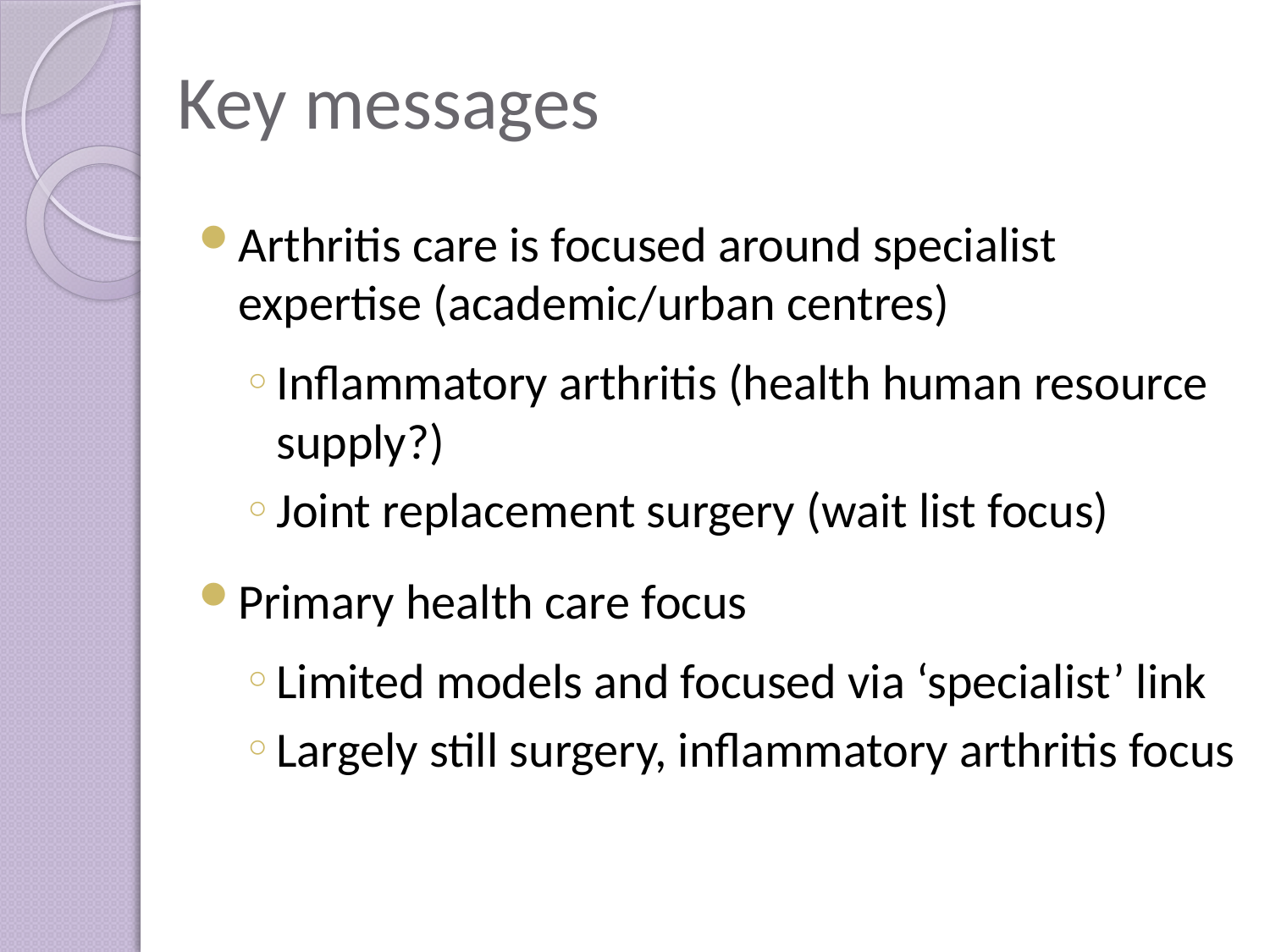

# Key messages
Arthritis care is focused around specialist expertise (academic/urban centres)
Inflammatory arthritis (health human resource supply?)
Joint replacement surgery (wait list focus)
Primary health care focus
Limited models and focused via ‘specialist’ link
Largely still surgery, inflammatory arthritis focus

## Slide 7
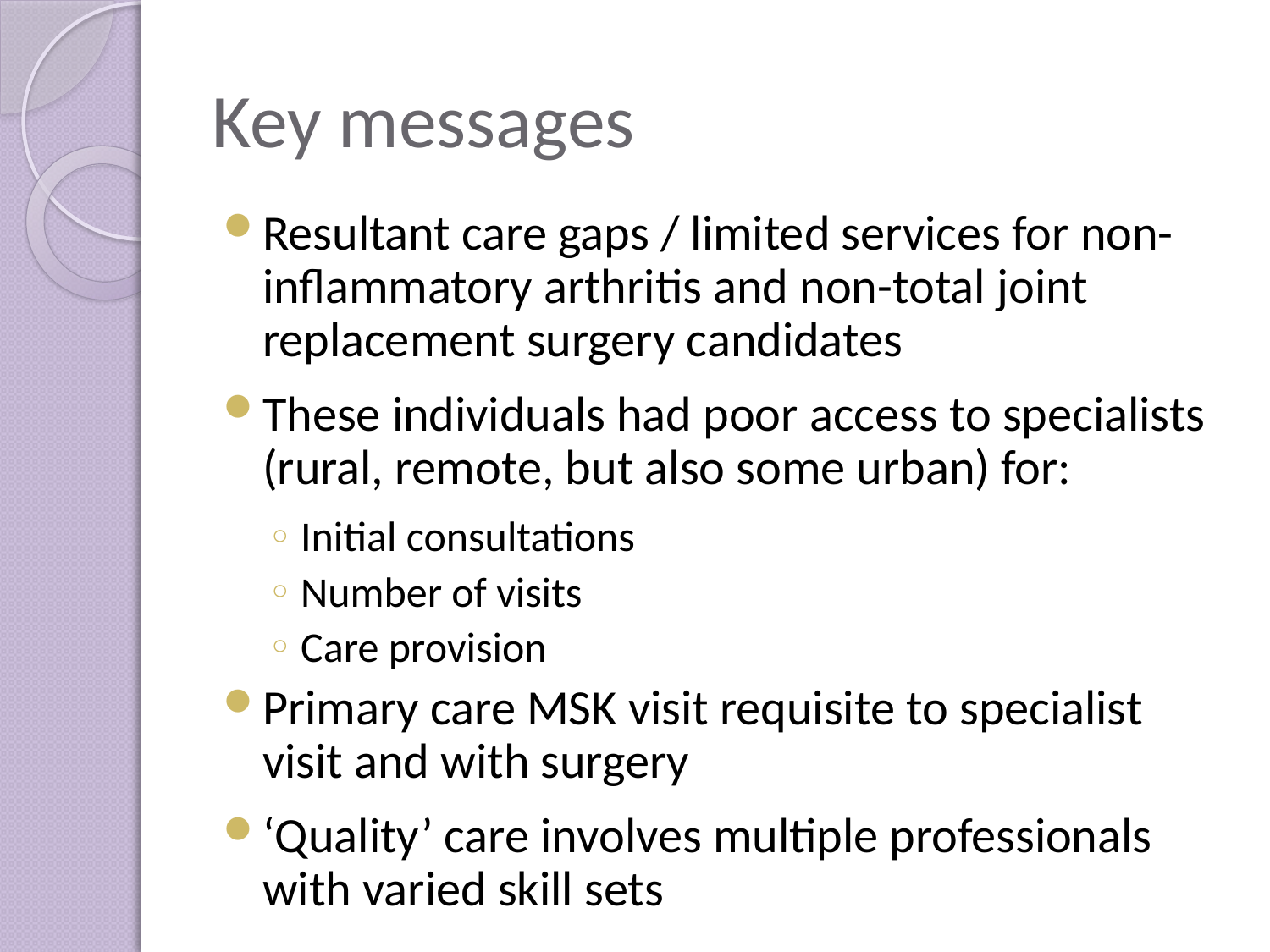

# Key messages
Resultant care gaps / limited services for non-inflammatory arthritis and non-total joint replacement surgery candidates
These individuals had poor access to specialists (rural, remote, but also some urban) for:
Initial consultations
Number of visits
Care provision
Primary care MSK visit requisite to specialist visit and with surgery
‘Quality’ care involves multiple professionals with varied skill sets

## Slide 8
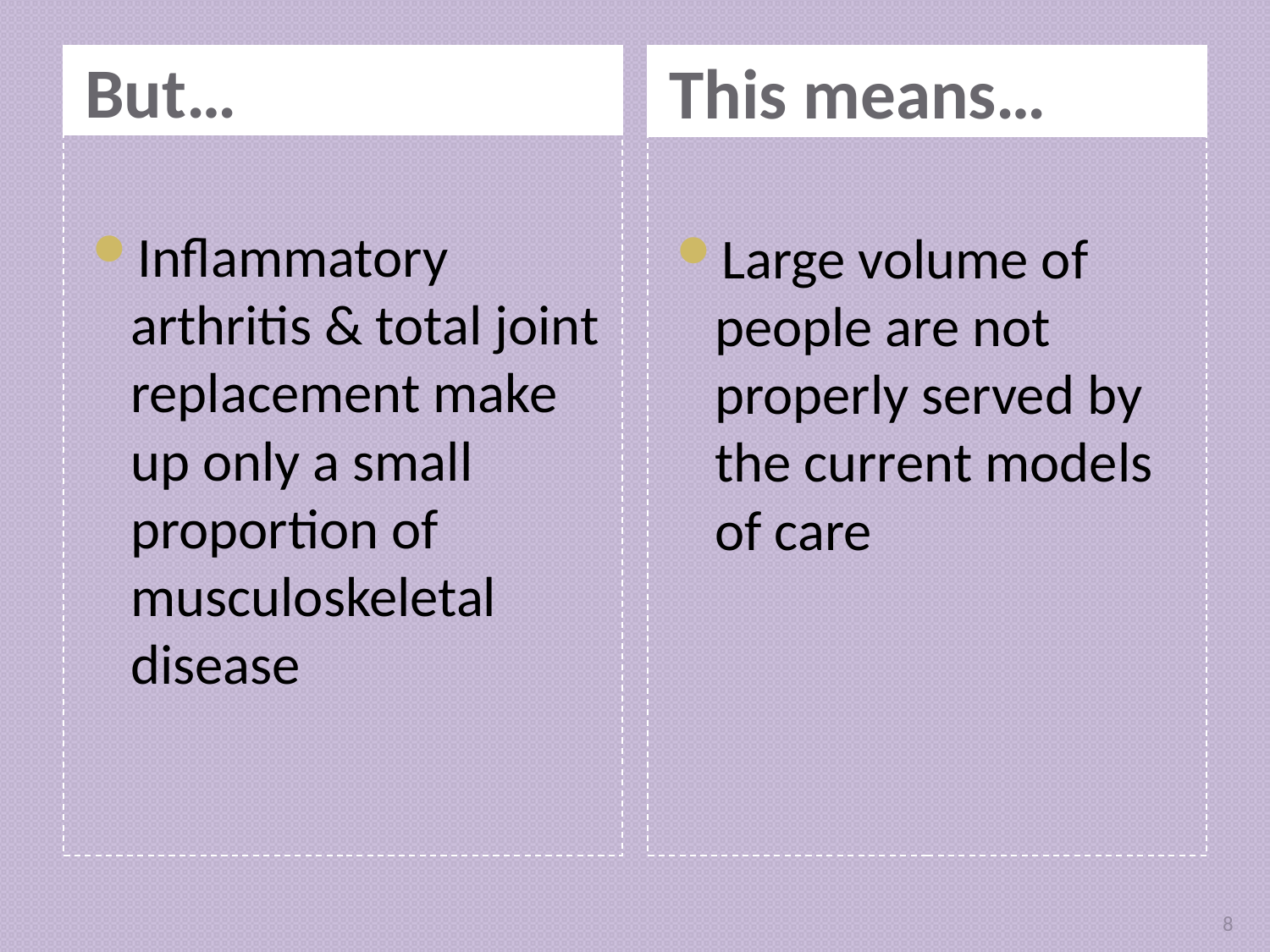

But…
This means…
Inflammatory arthritis & total joint replacement make up only a small proportion of musculoskeletal disease
Large volume of people are not properly served by the current models of care
8

## Slide 9
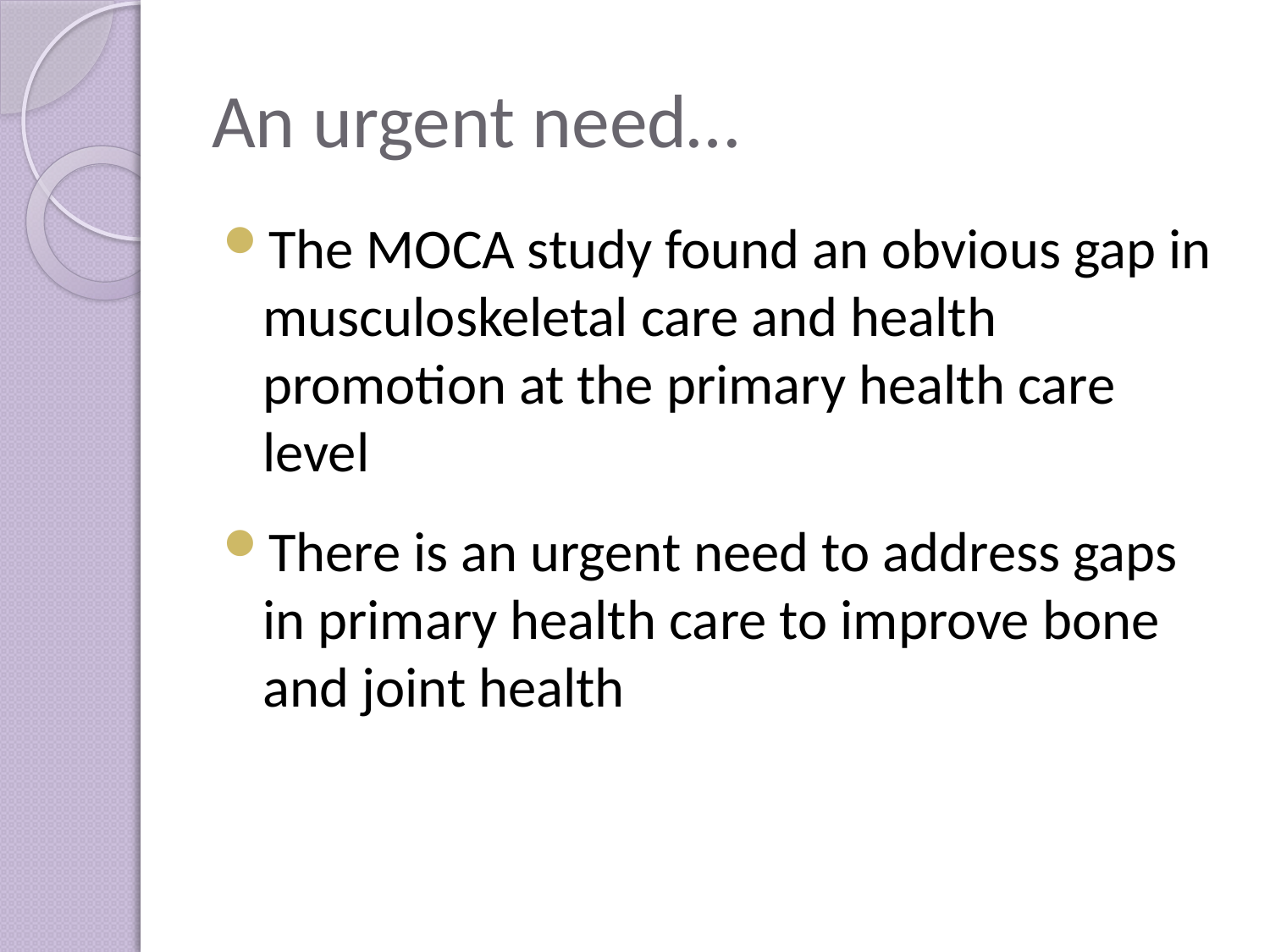

# An urgent need…
The MOCA study found an obvious gap in musculoskeletal care and health promotion at the primary health care level
There is an urgent need to address gaps in primary health care to improve bone and joint health

## Slide 10
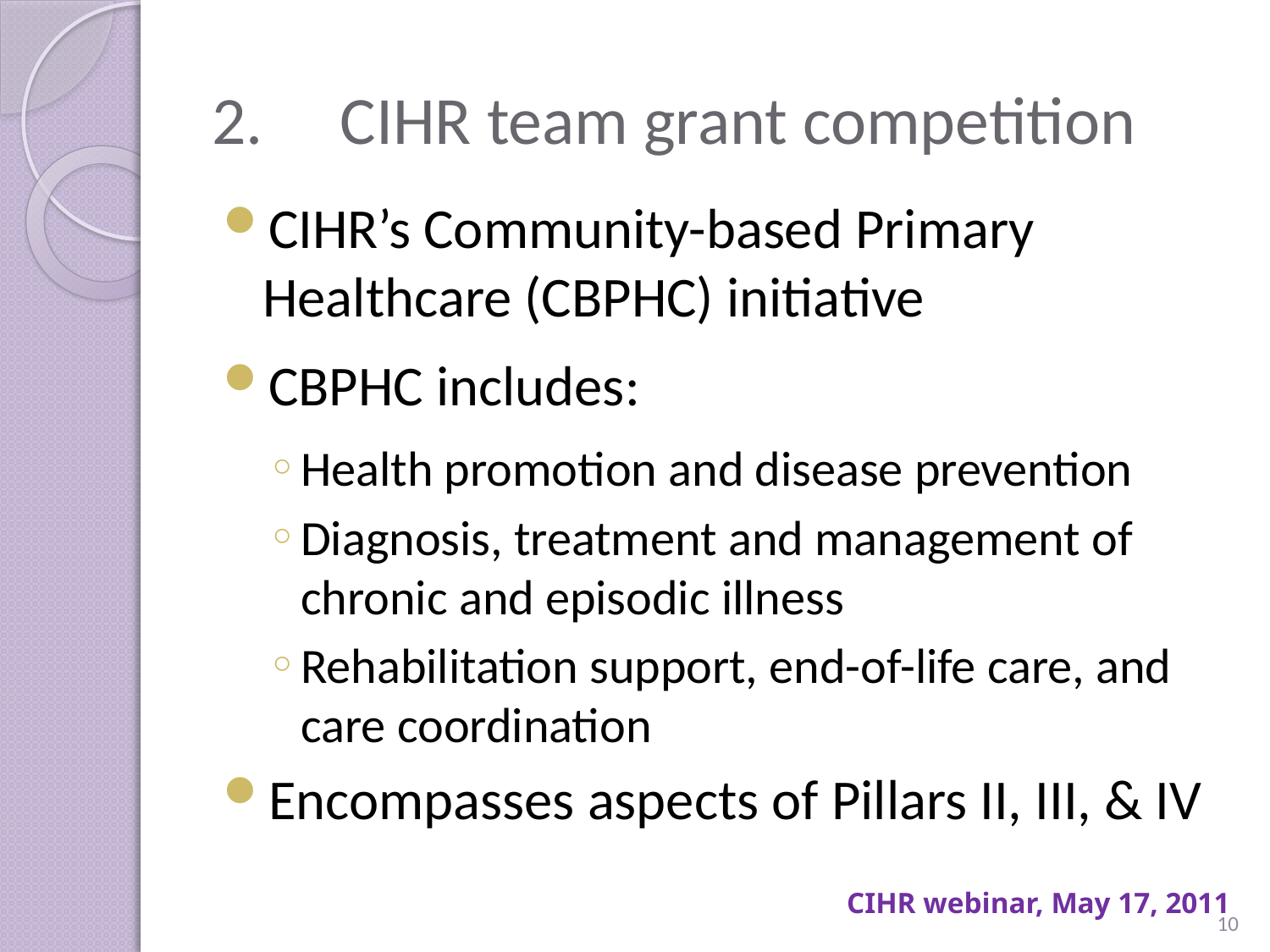

# 2.	 CIHR team grant competition
CIHR’s Community-based Primary Healthcare (CBPHC) initiative
CBPHC includes:
Health promotion and disease prevention
Diagnosis, treatment and management of chronic and episodic illness
Rehabilitation support, end-of-life care, and care coordination
Encompasses aspects of Pillars II, III, & IV
10
CIHR webinar, May 17, 2011

## Slide 11
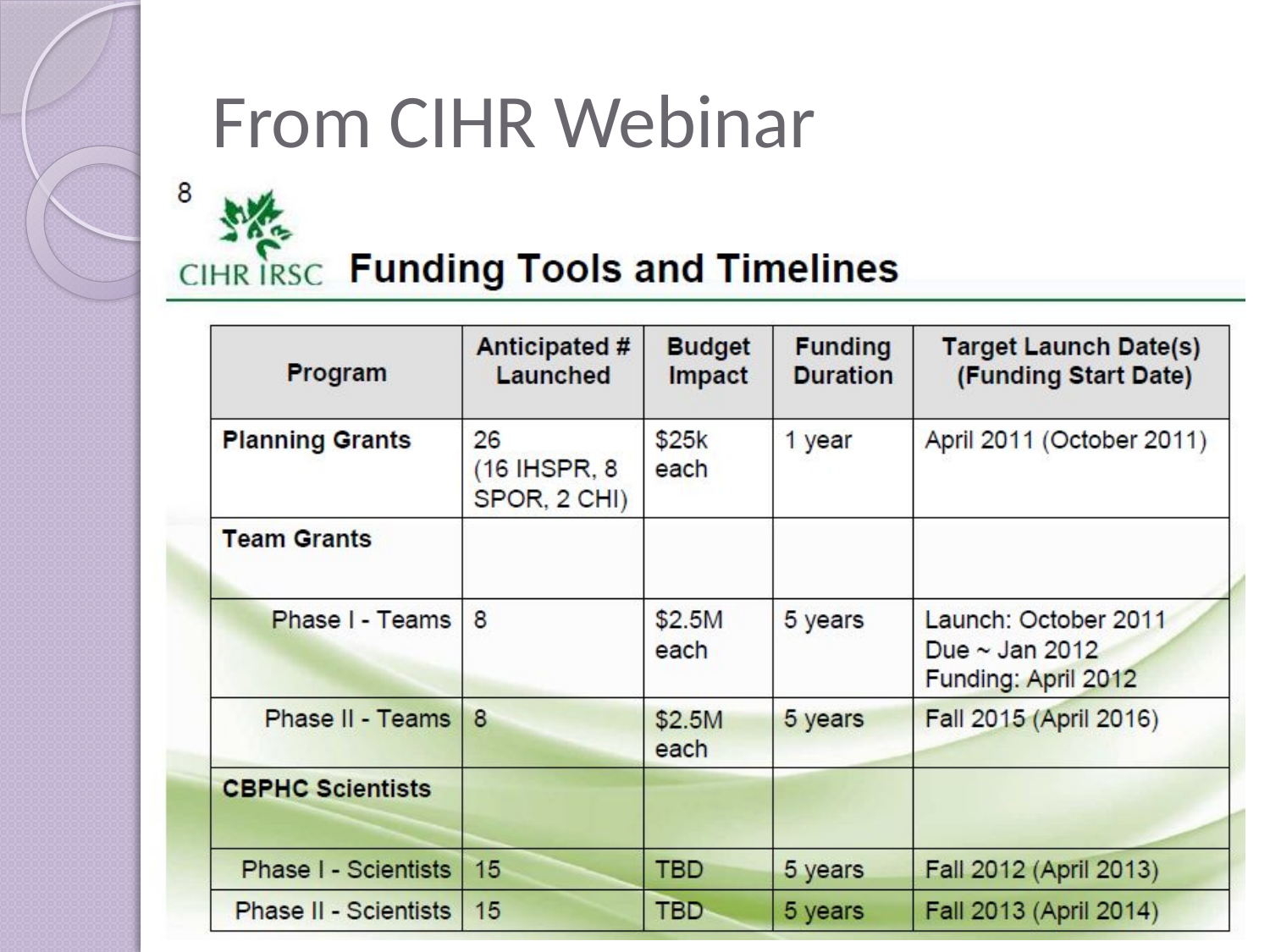

# From CIHR Webinar
11

## Slide 12
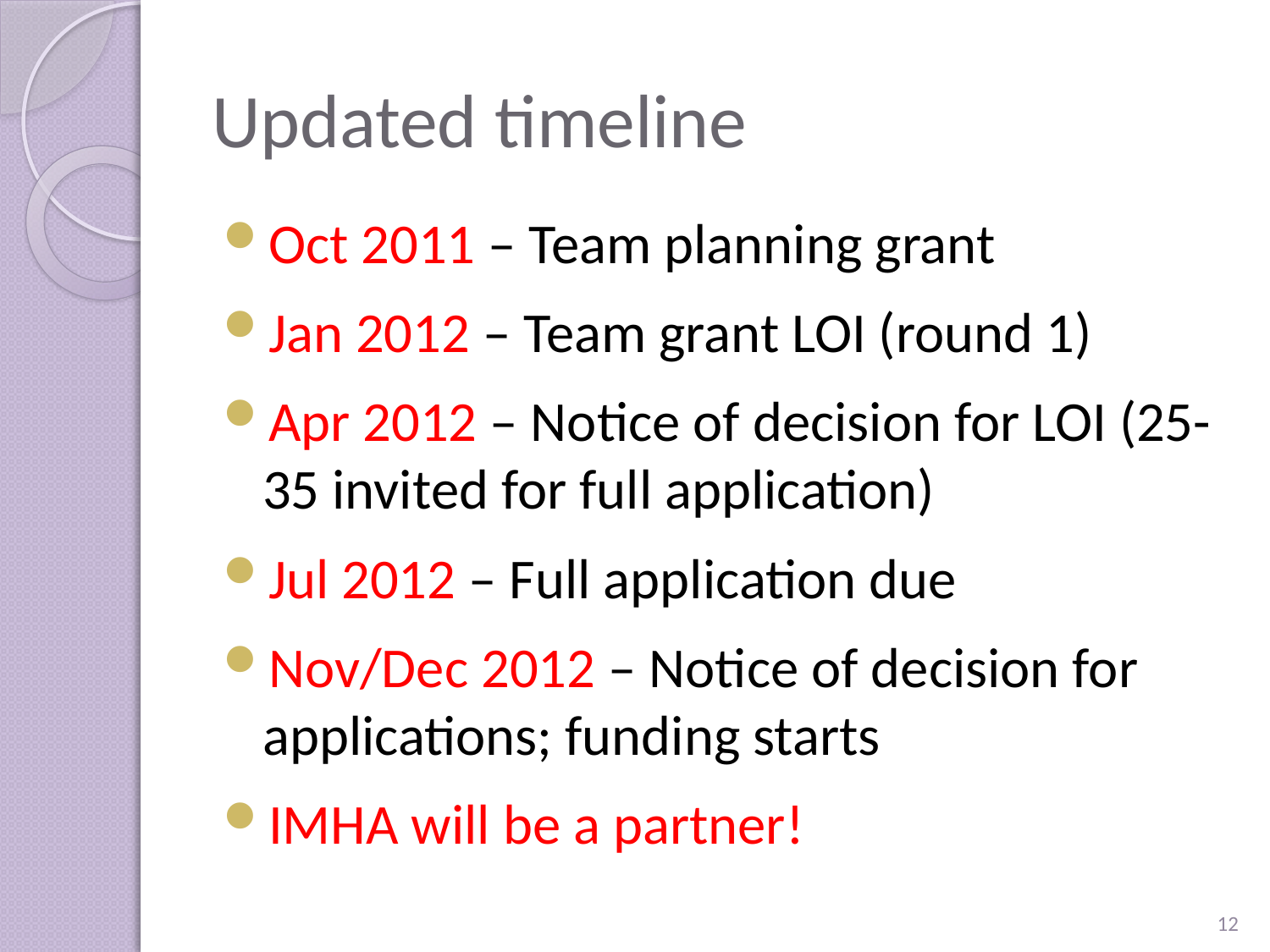

# Updated timeline
Oct 2011 – Team planning grant
Jan 2012 – Team grant LOI (round 1)
Apr 2012 – Notice of decision for LOI (25-35 invited for full application)
Jul 2012 – Full application due
Nov/Dec 2012 – Notice of decision for applications; funding starts
IMHA will be a partner!
12

## Slide 13
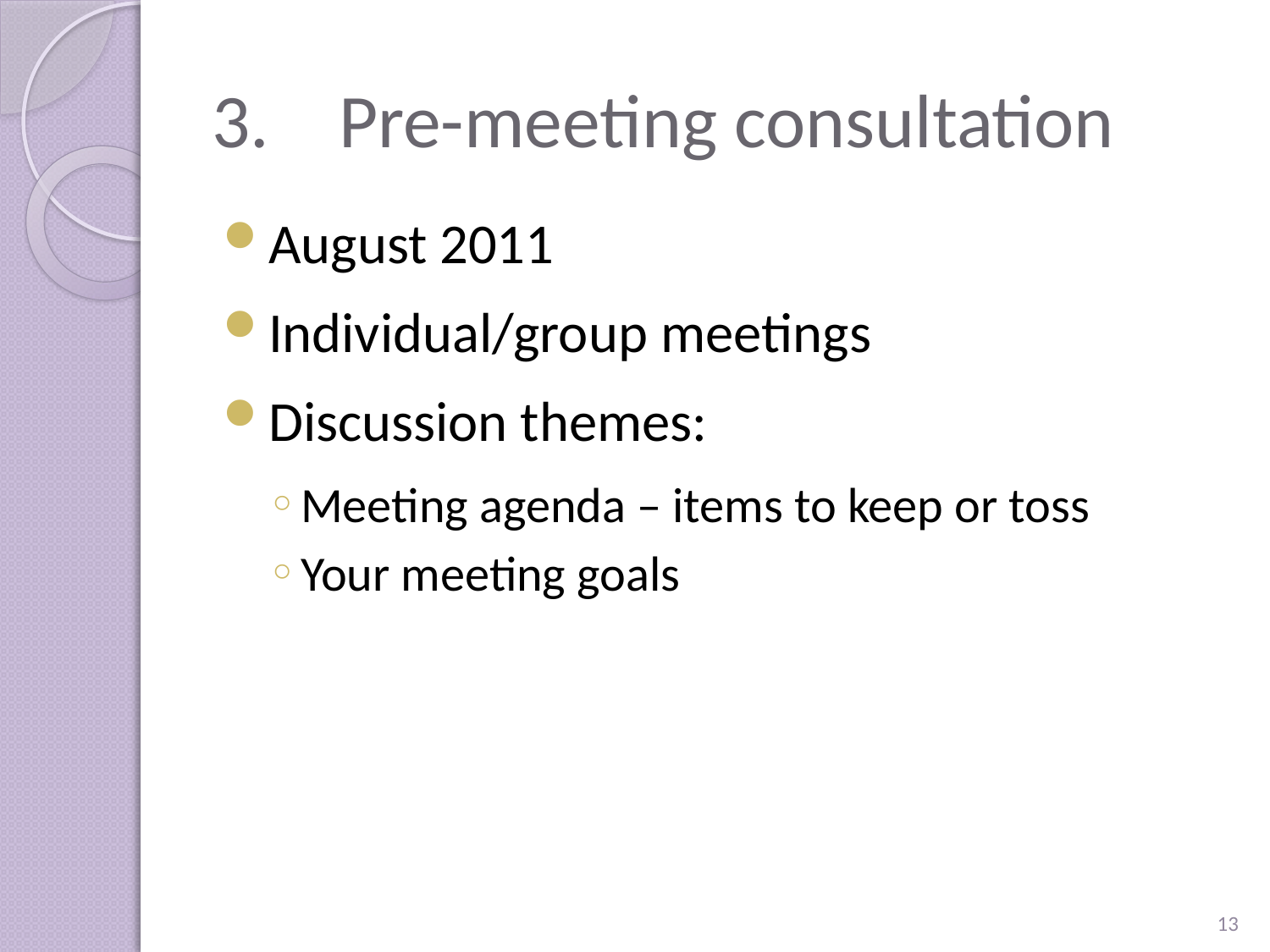

# 3.	Pre-meeting consultation
August 2011
Individual/group meetings
Discussion themes:
Meeting agenda – items to keep or toss
Your meeting goals
13

## Slide 14
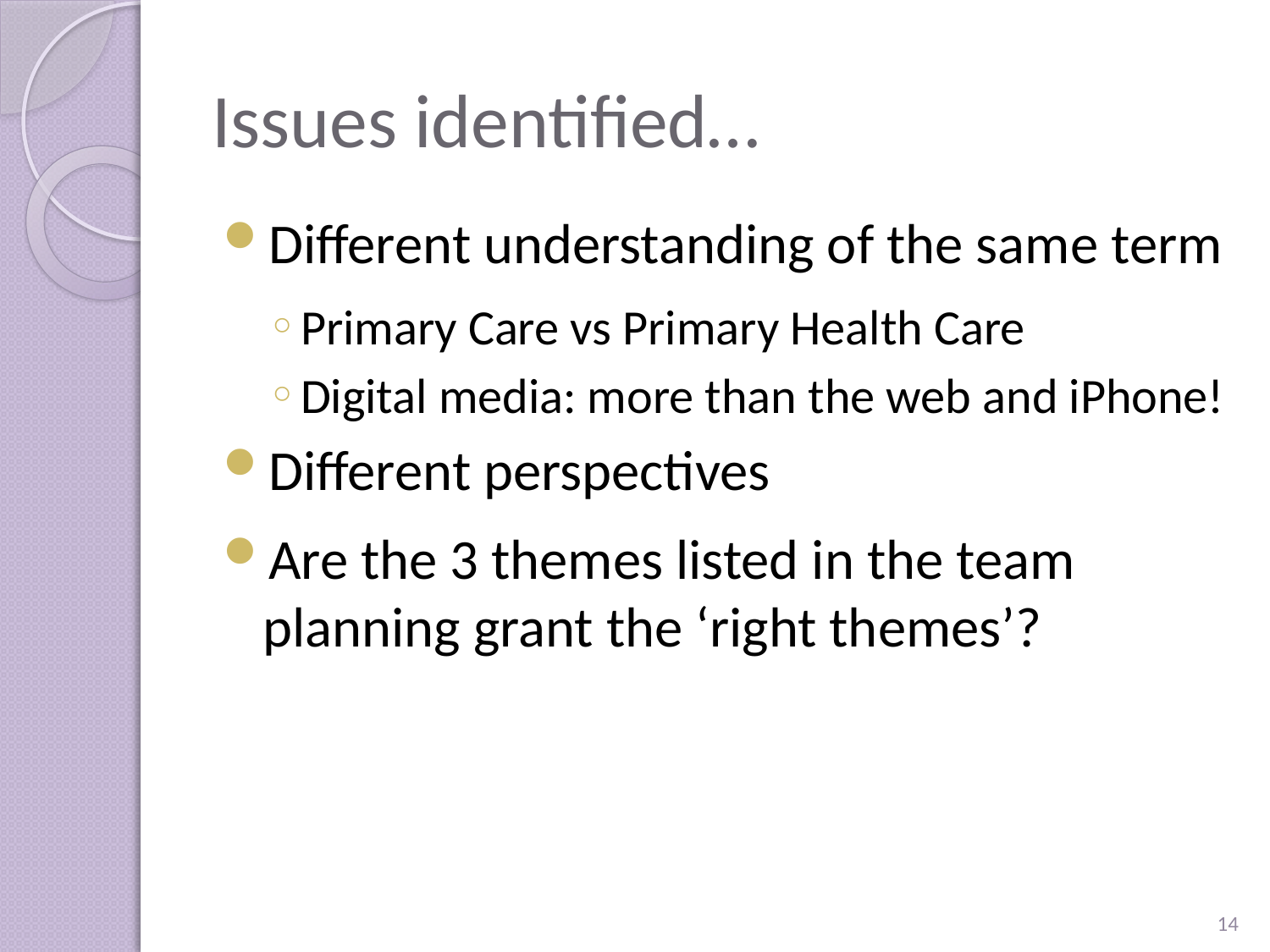

# Issues identified…
Different understanding of the same term
Primary Care vs Primary Health Care
Digital media: more than the web and iPhone!
Different perspectives
Are the 3 themes listed in the team planning grant the ‘right themes’?
14

## Slide 15
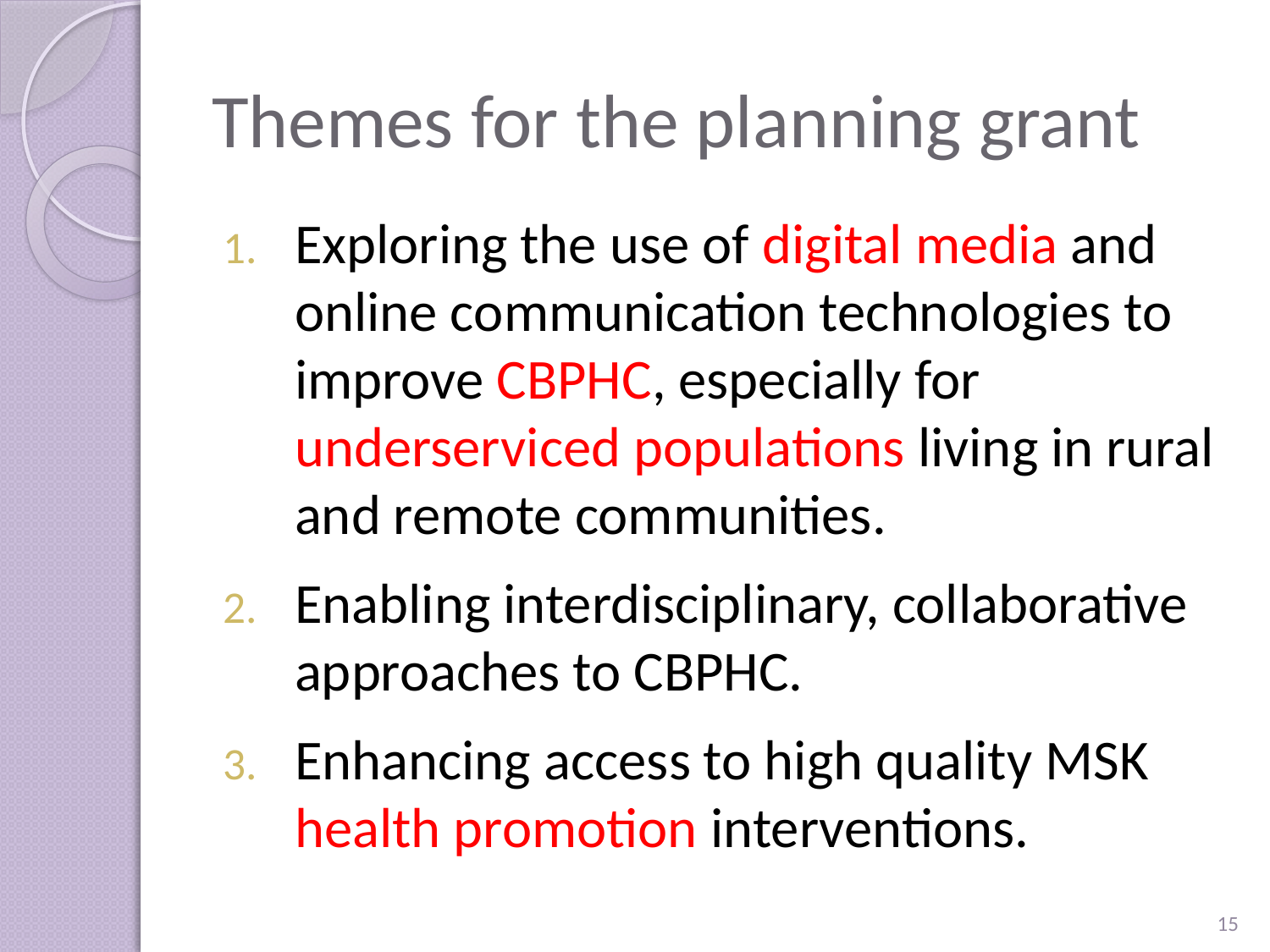

# Themes for the planning grant
Exploring the use of digital media and online communication technologies to improve CBPHC, especially for underserviced populations living in rural and remote communities.
Enabling interdisciplinary, collaborative approaches to CBPHC.
Enhancing access to high quality MSK health promotion interventions.
15

## Slide 16
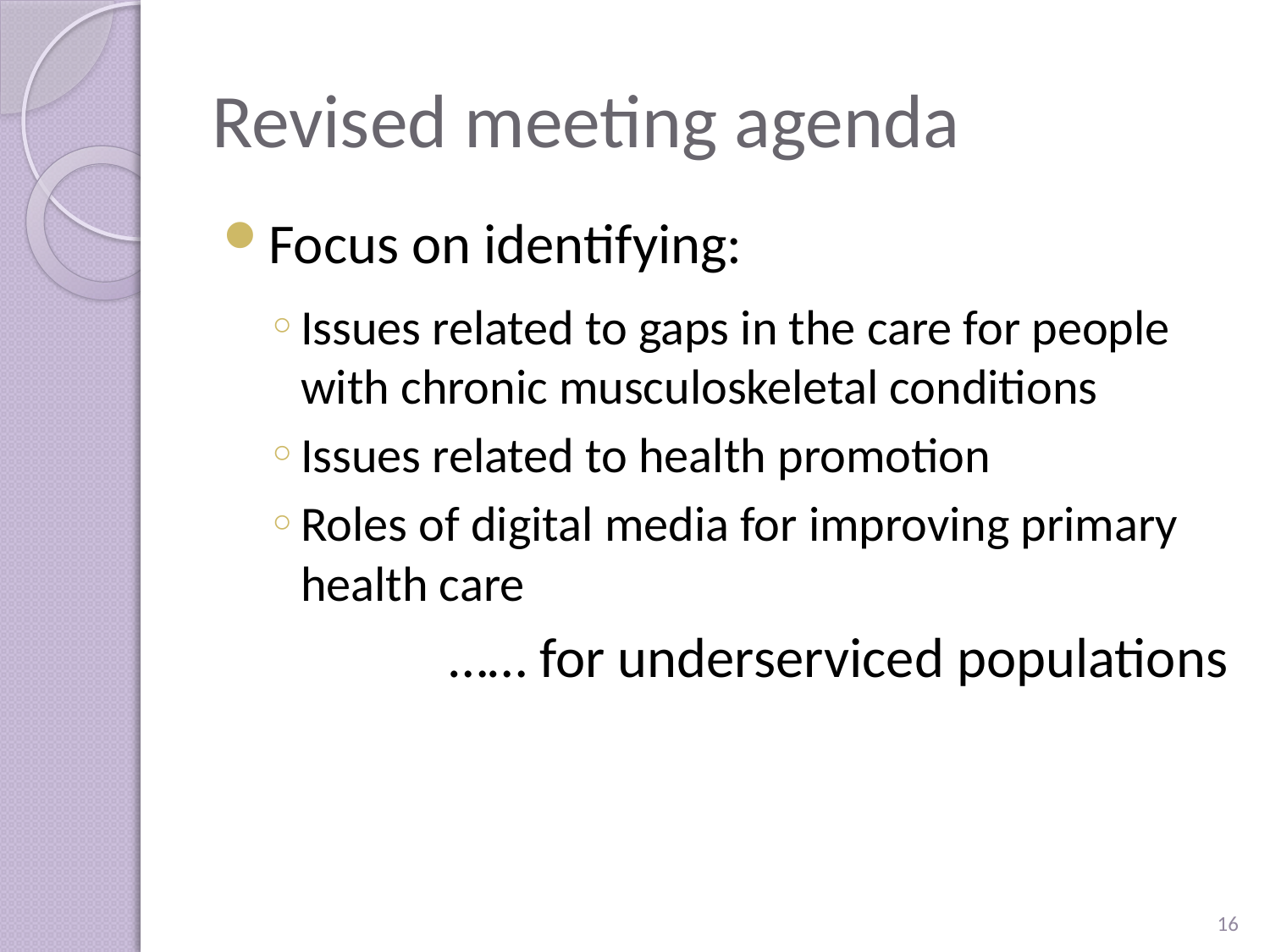

# Revised meeting agenda
Focus on identifying:
Issues related to gaps in the care for people with chronic musculoskeletal conditions
Issues related to health promotion
Roles of digital media for improving primary health care
…… for underserviced populations
16

## Slide 17
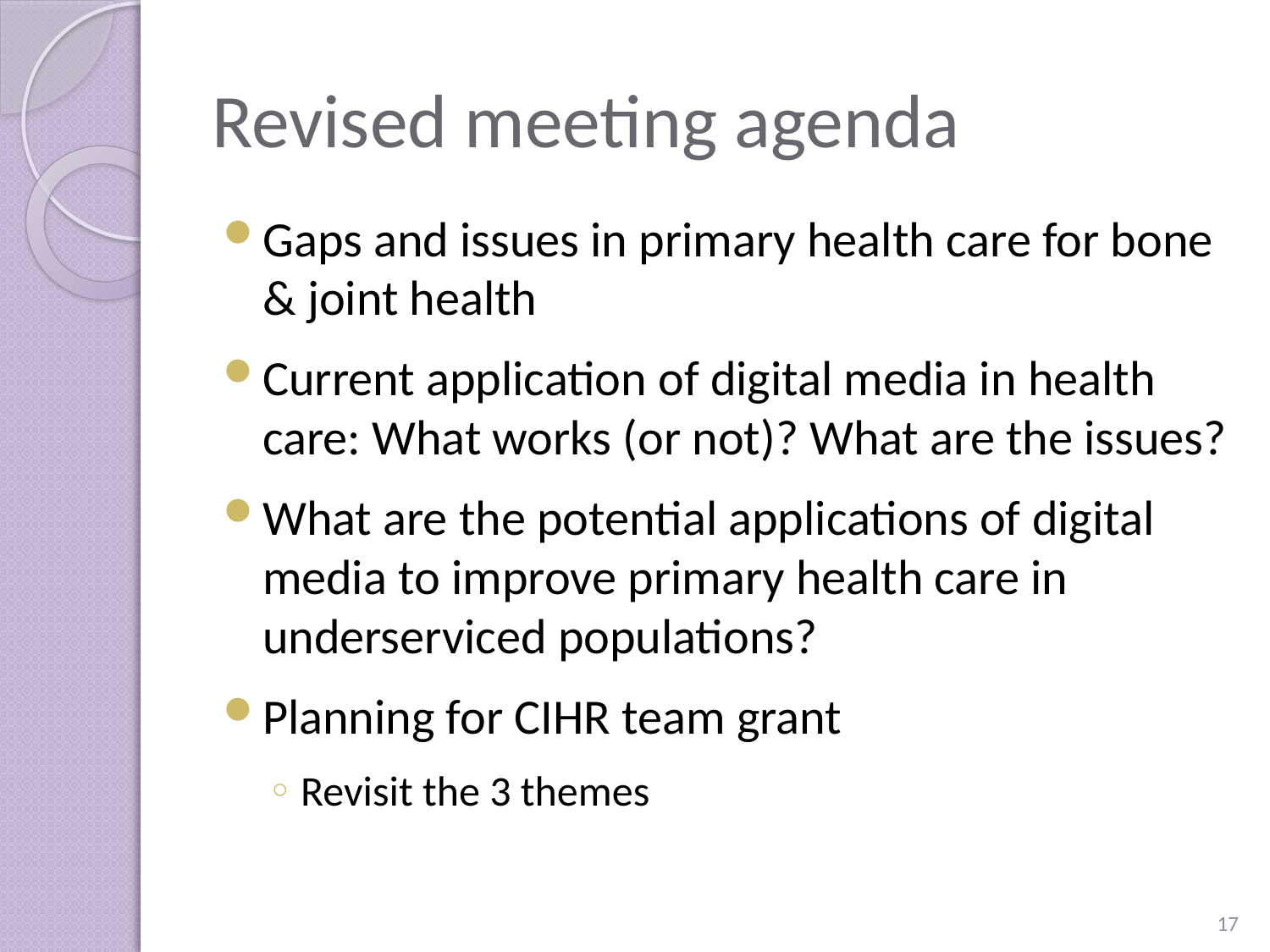

# Revised meeting agenda
Gaps and issues in primary health care for bone & joint health
Current application of digital media in health care: What works (or not)? What are the issues?
What are the potential applications of digital media to improve primary health care in underserviced populations?
Planning for CIHR team grant
Revisit the 3 themes
17

## Slide 18
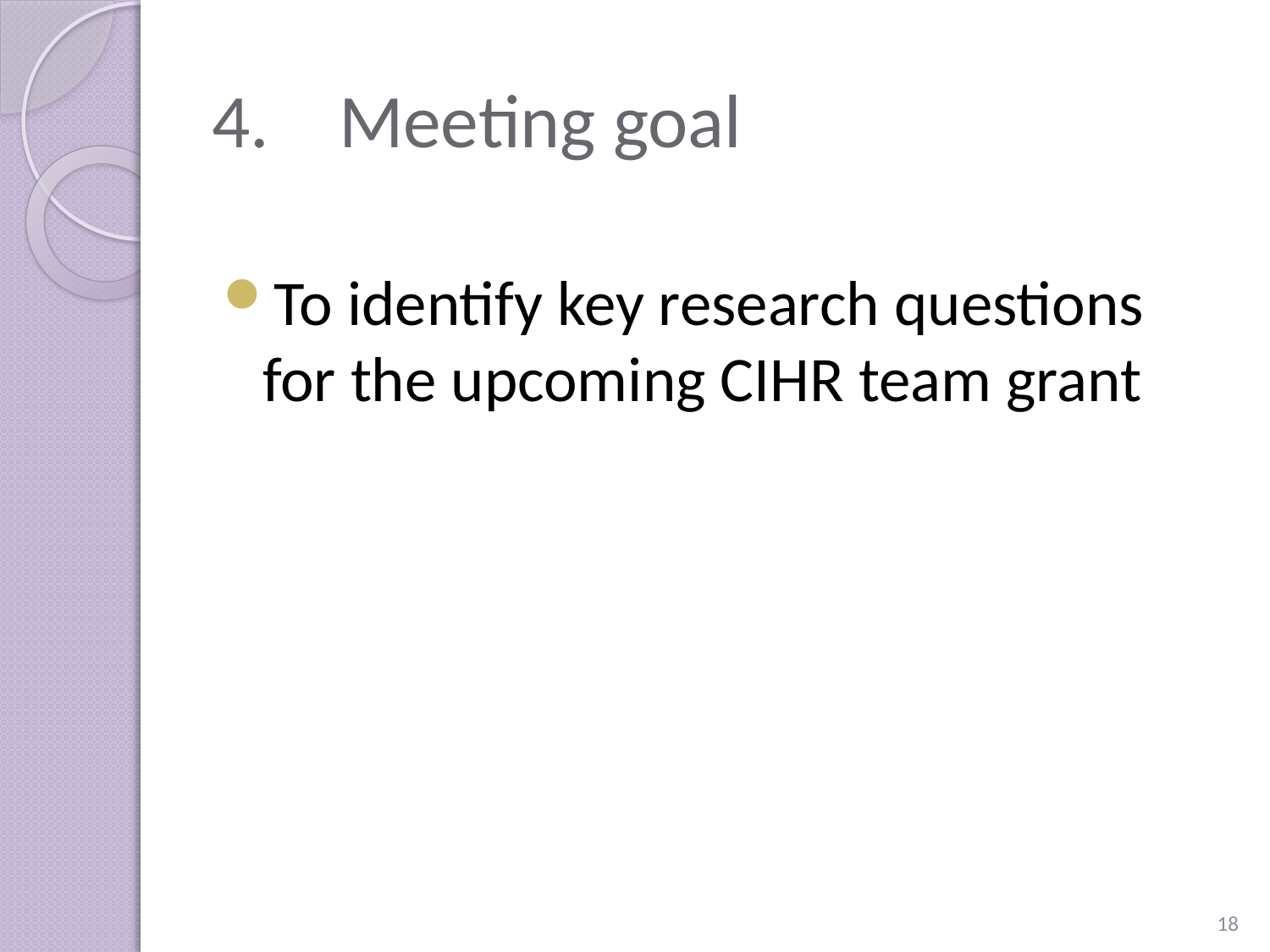

# 4.	Meeting goal
To identify key research questions for the upcoming CIHR team grant
18

## Slide 19
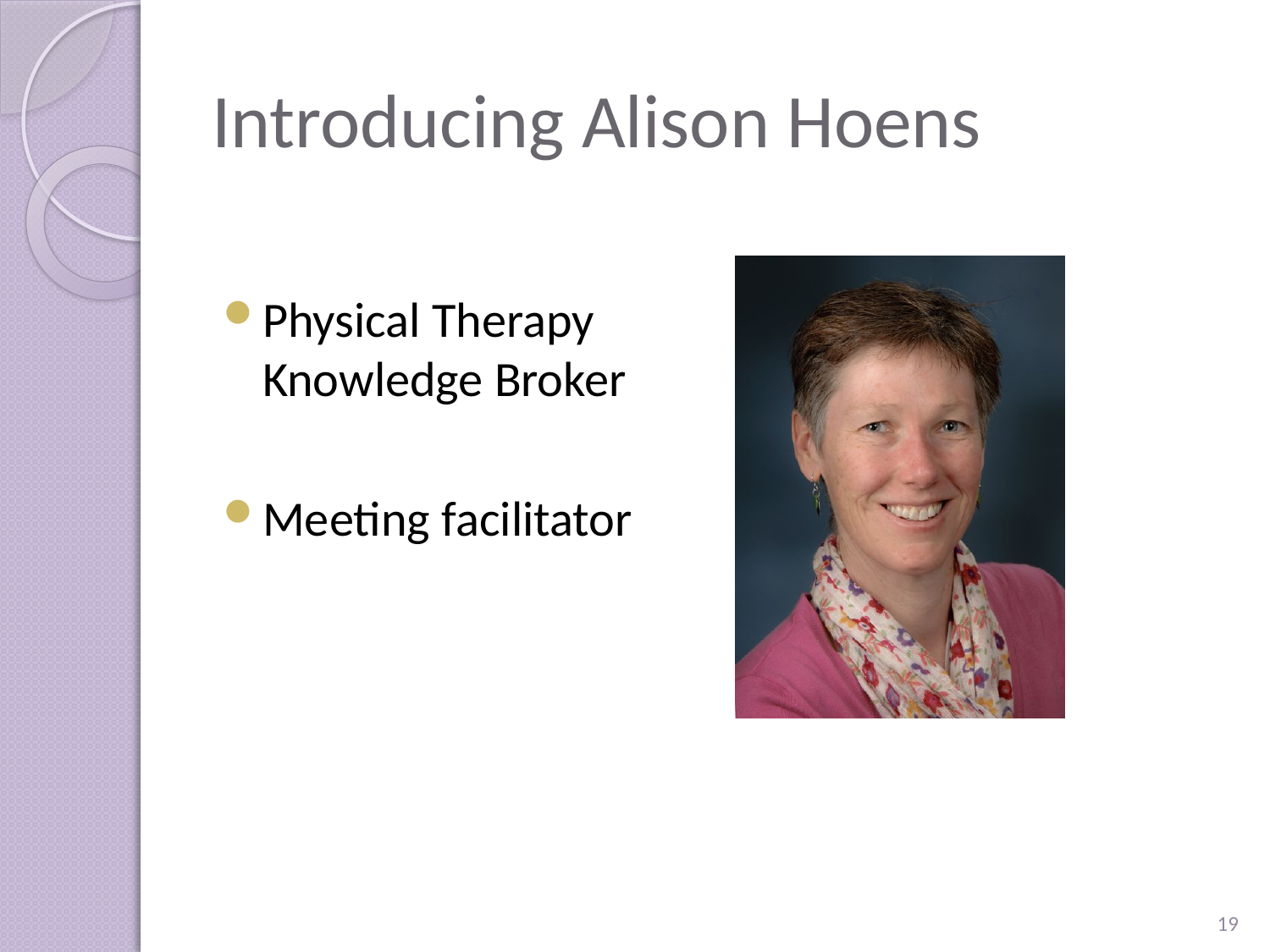

# Introducing Alison Hoens
Physical Therapy Knowledge Broker
Meeting facilitator
19

## Slide 20
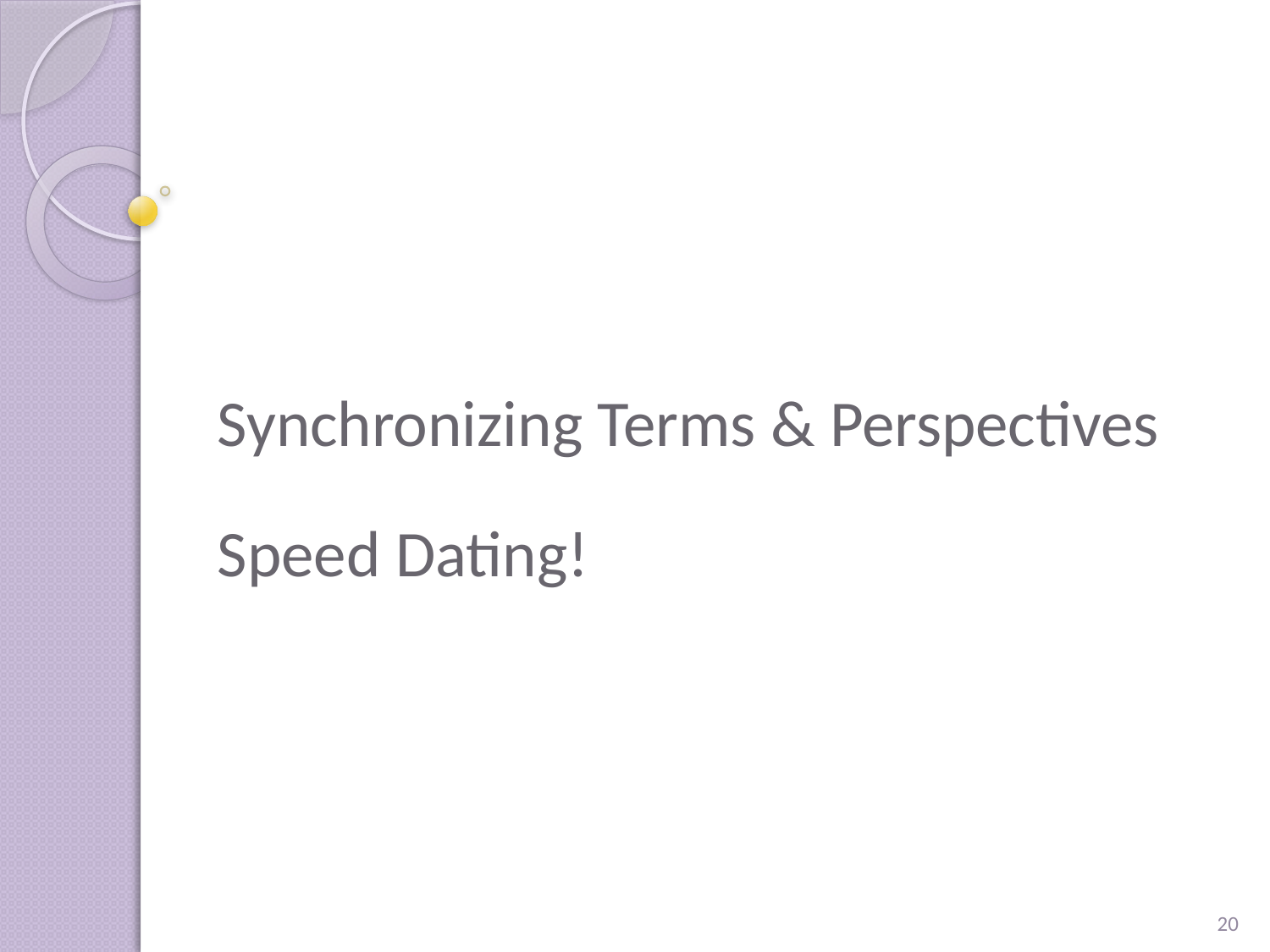

# Synchronizing Terms & Perspectives
Speed Dating!
20

## Slide 21
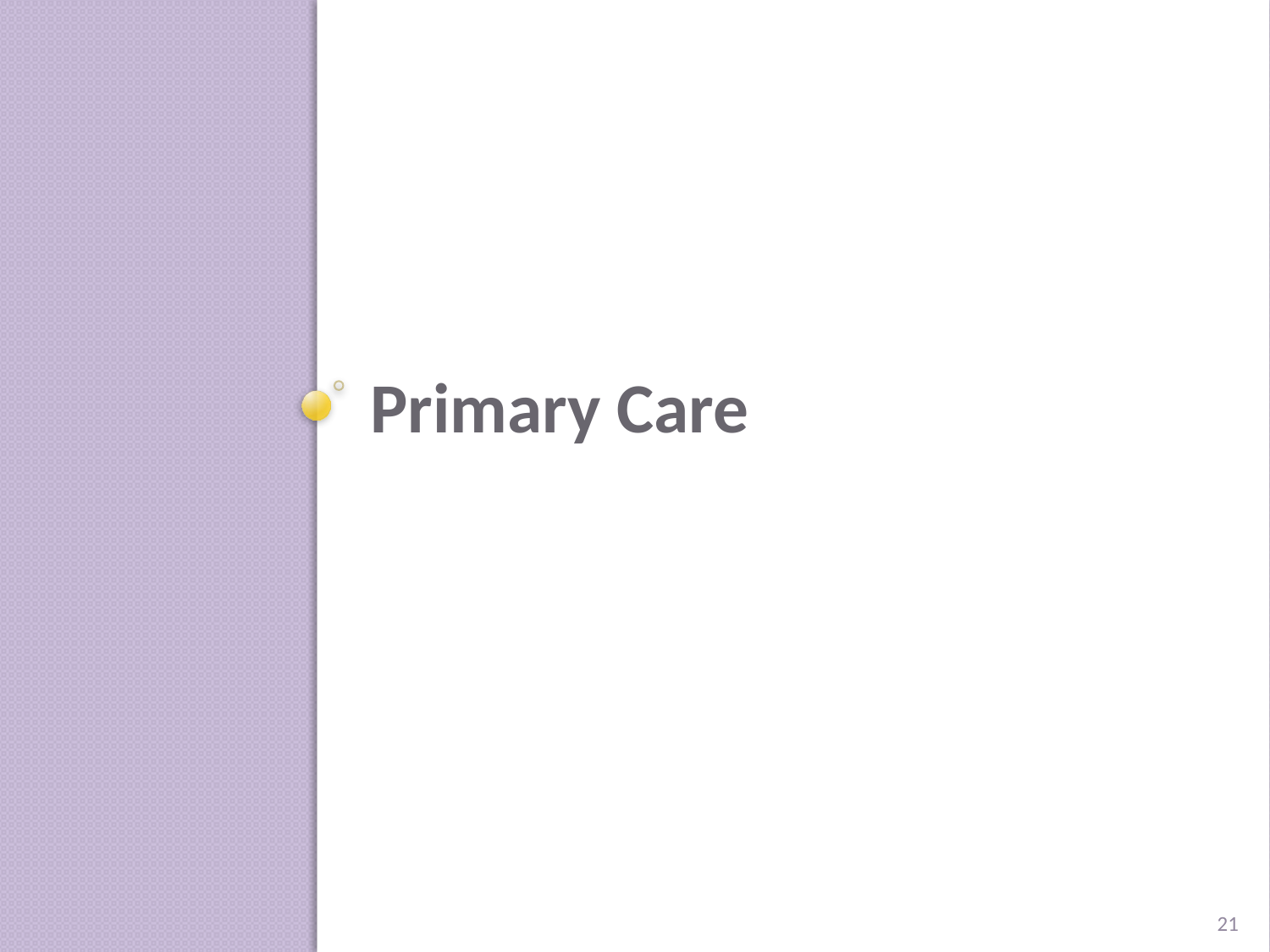

# Primary Care
21

## Slide 22
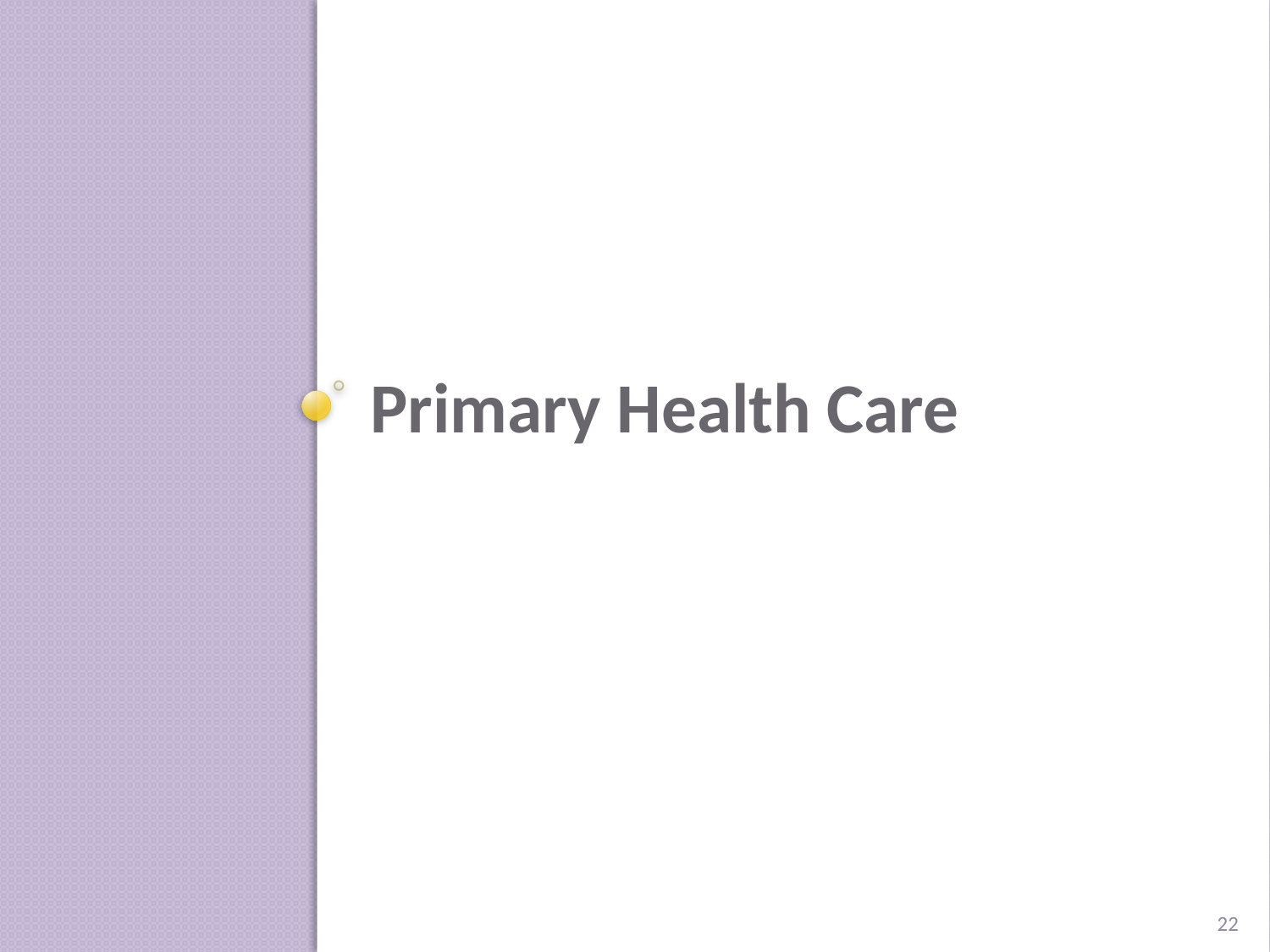

# Primary Health Care
22

## Slide 23
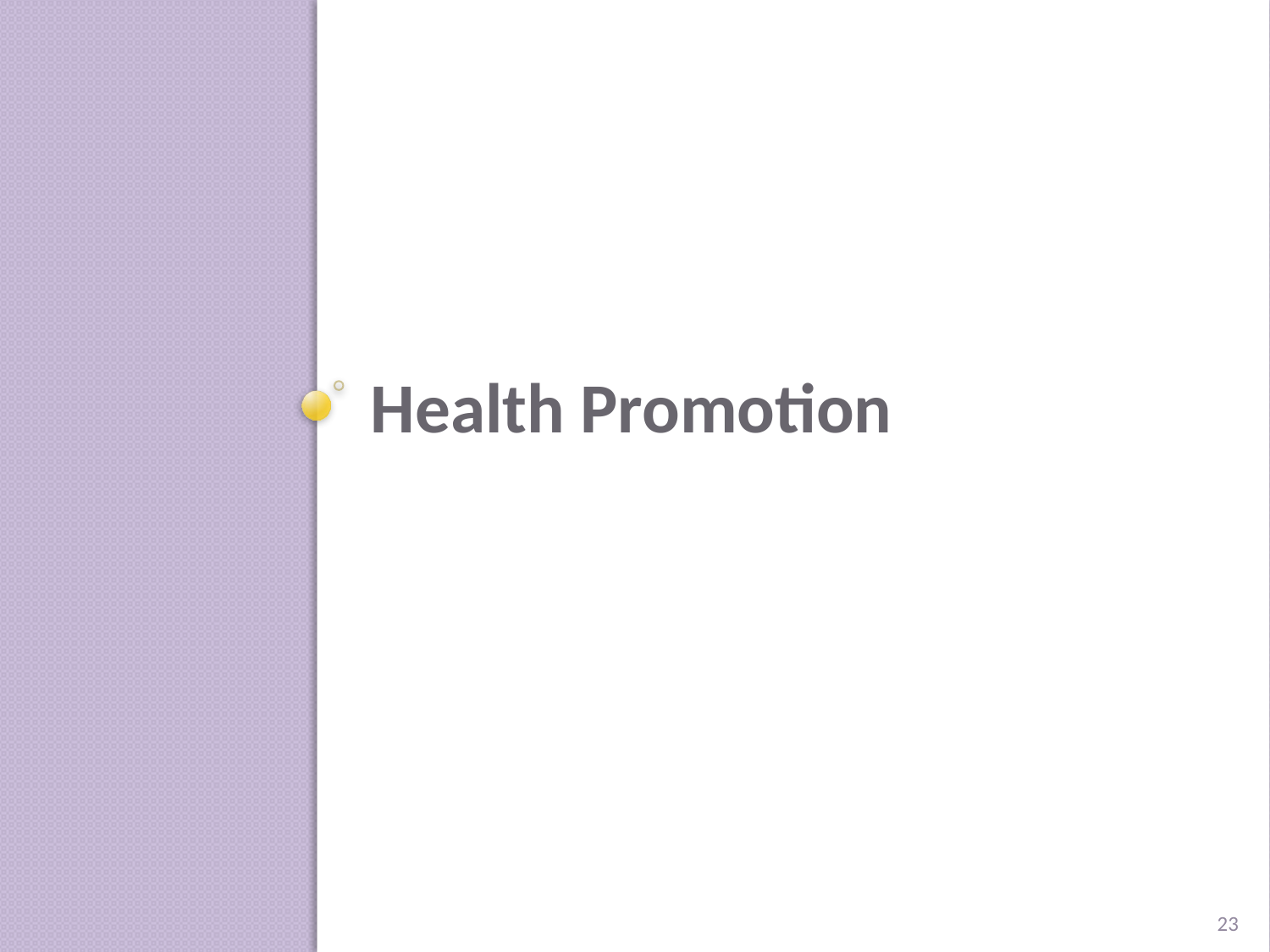

# Health Promotion
23

## Slide 24
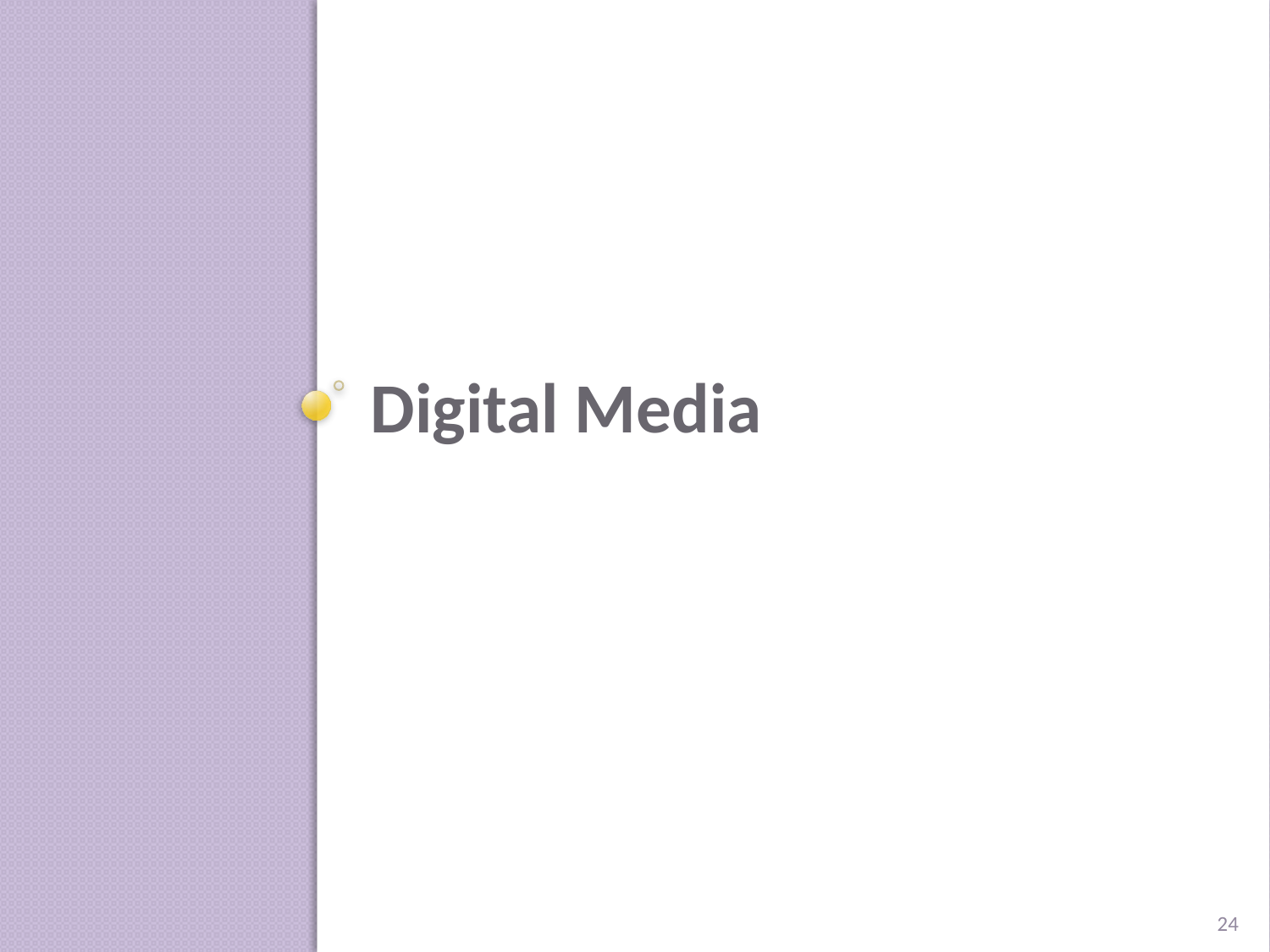

# Digital Media
24

## Slide 25
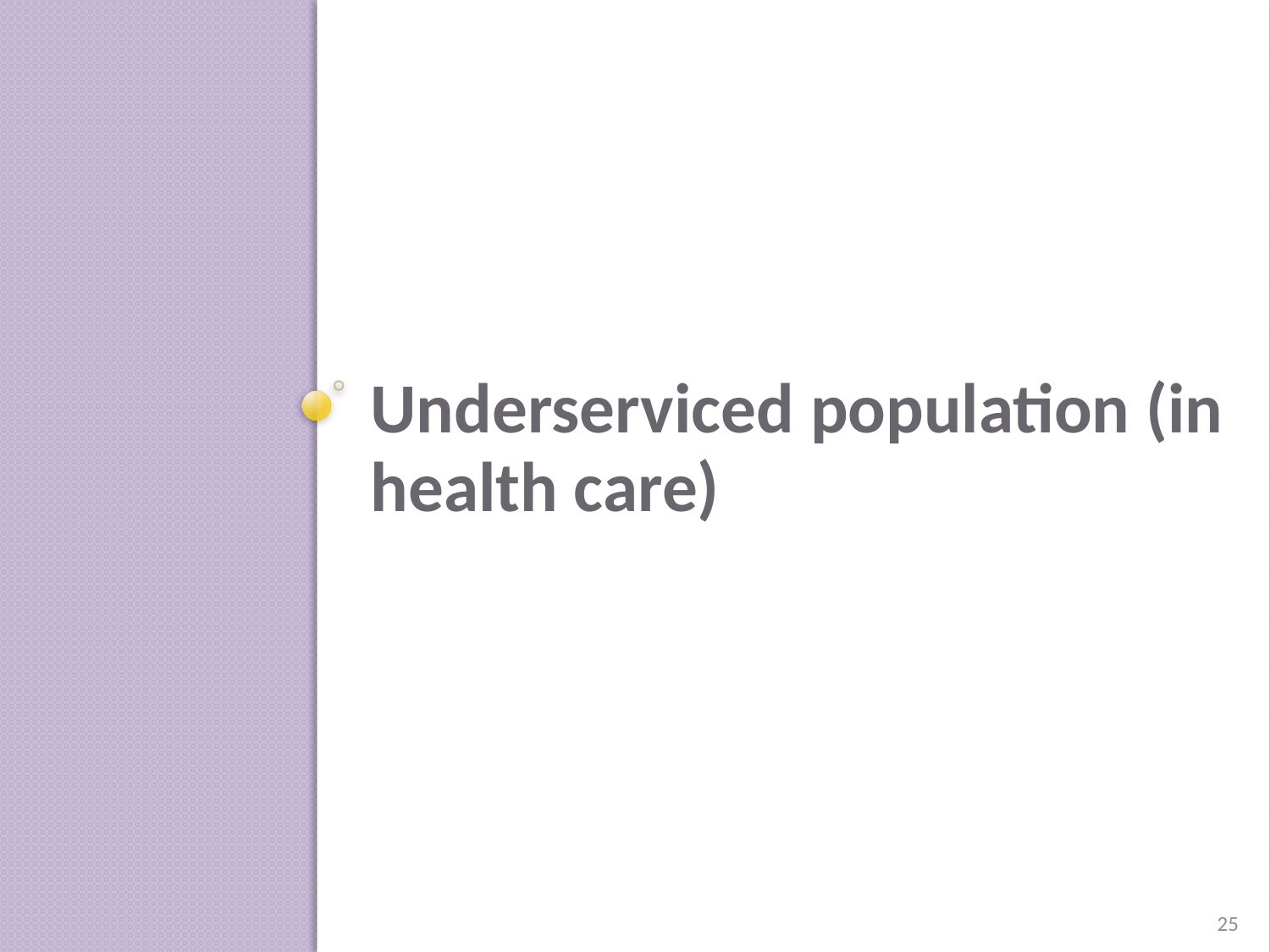

# Underserviced population (in health care)
25

## Slide 26
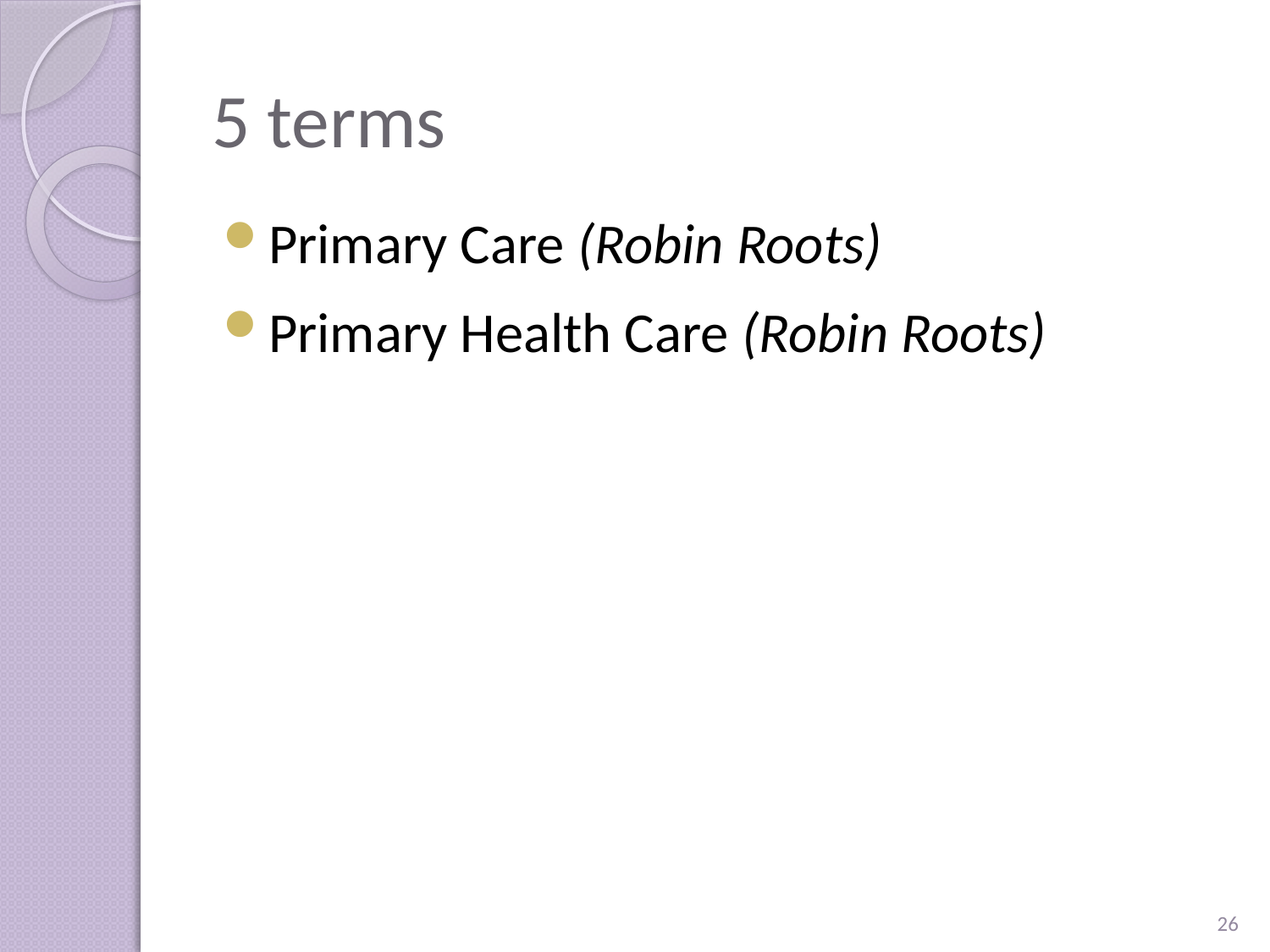

# 5 terms
Primary Care (Robin Roots)
Primary Health Care (Robin Roots)
26

## Slide 27
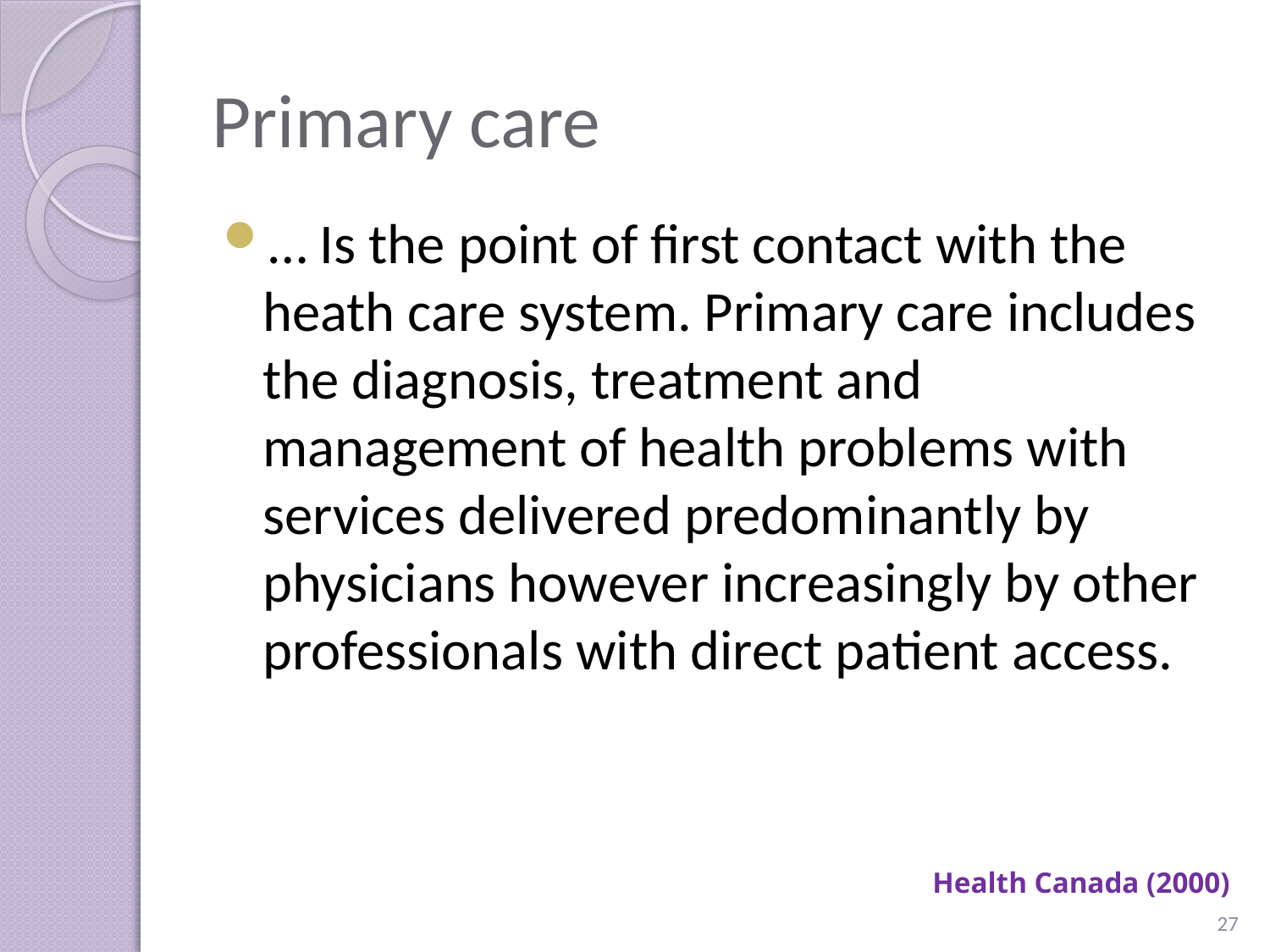

# Primary care
… Is the point of first contact with the heath care system. Primary care includes the diagnosis, treatment and management of health problems with services delivered predominantly by physicians however increasingly by other professionals with direct patient access.
Health Canada (2000)
27

## Slide 28
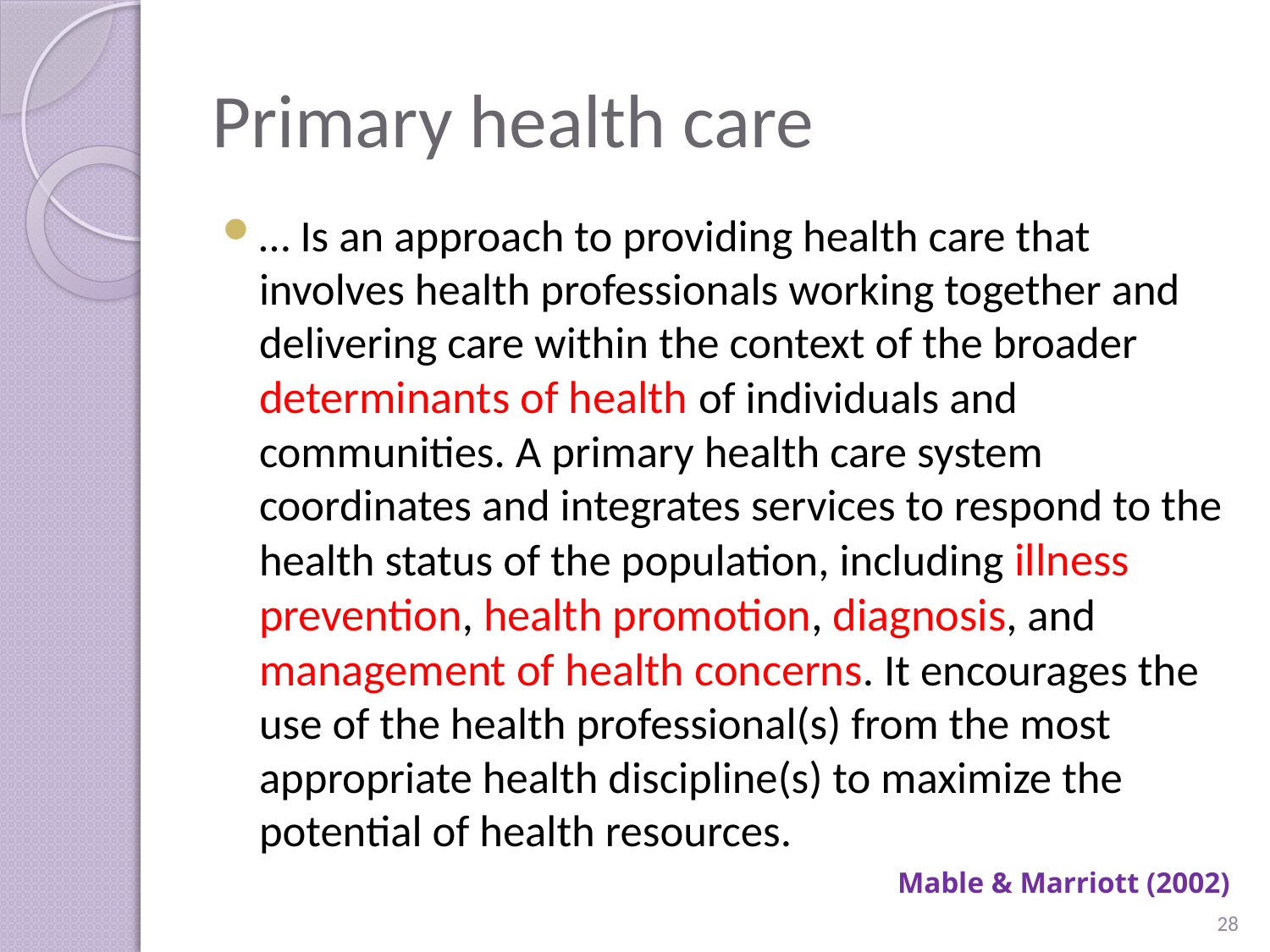

# Primary health care
… Is an approach to providing health care that involves health professionals working together and delivering care within the context of the broader determinants of health of individuals and communities. A primary health care system coordinates and integrates services to respond to the health status of the population, including illness prevention, health promotion, diagnosis, and management of health concerns. It encourages the use of the health professional(s) from the most appropriate health discipline(s) to maximize the potential of health resources.
Mable & Marriott (2002)
28

## Slide 29
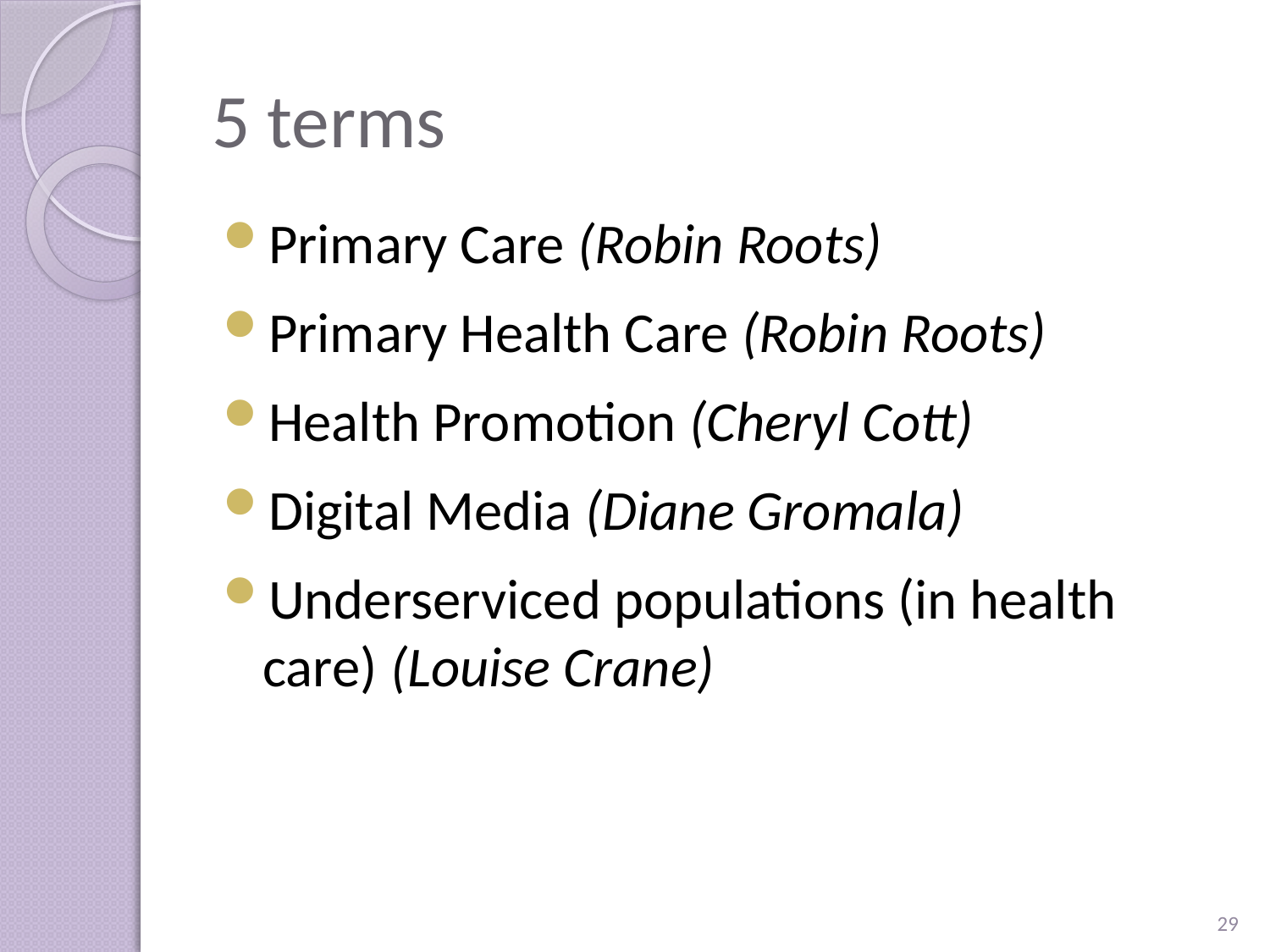

# 5 terms
Primary Care (Robin Roots)
Primary Health Care (Robin Roots)
Health Promotion (Cheryl Cott)
Digital Media (Diane Gromala)
Underserviced populations (in health care) (Louise Crane)
29

## Slide 30
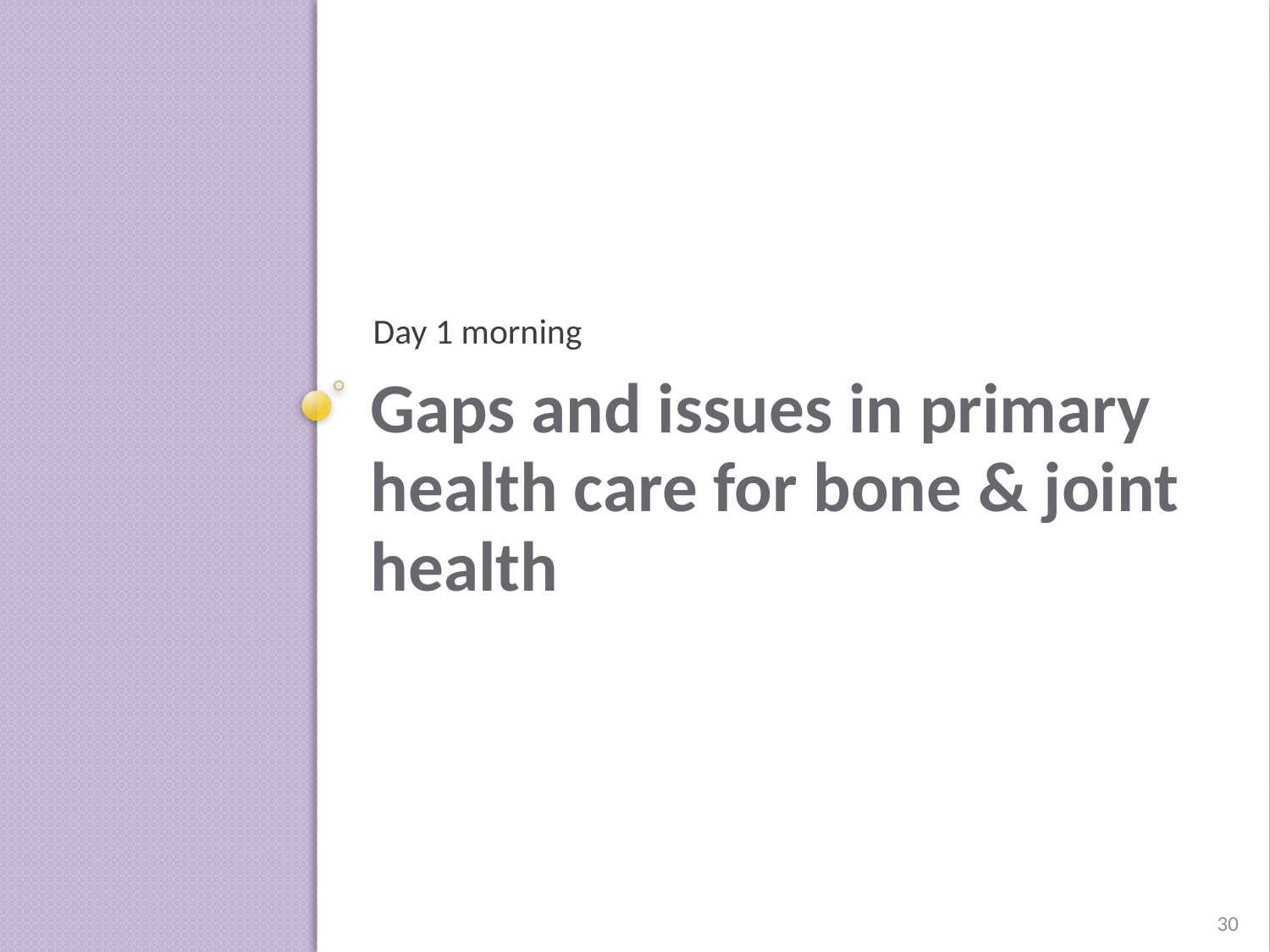

Day 1 morning
# Gaps and issues in primary health care for bone & joint health
30

## Slide 31
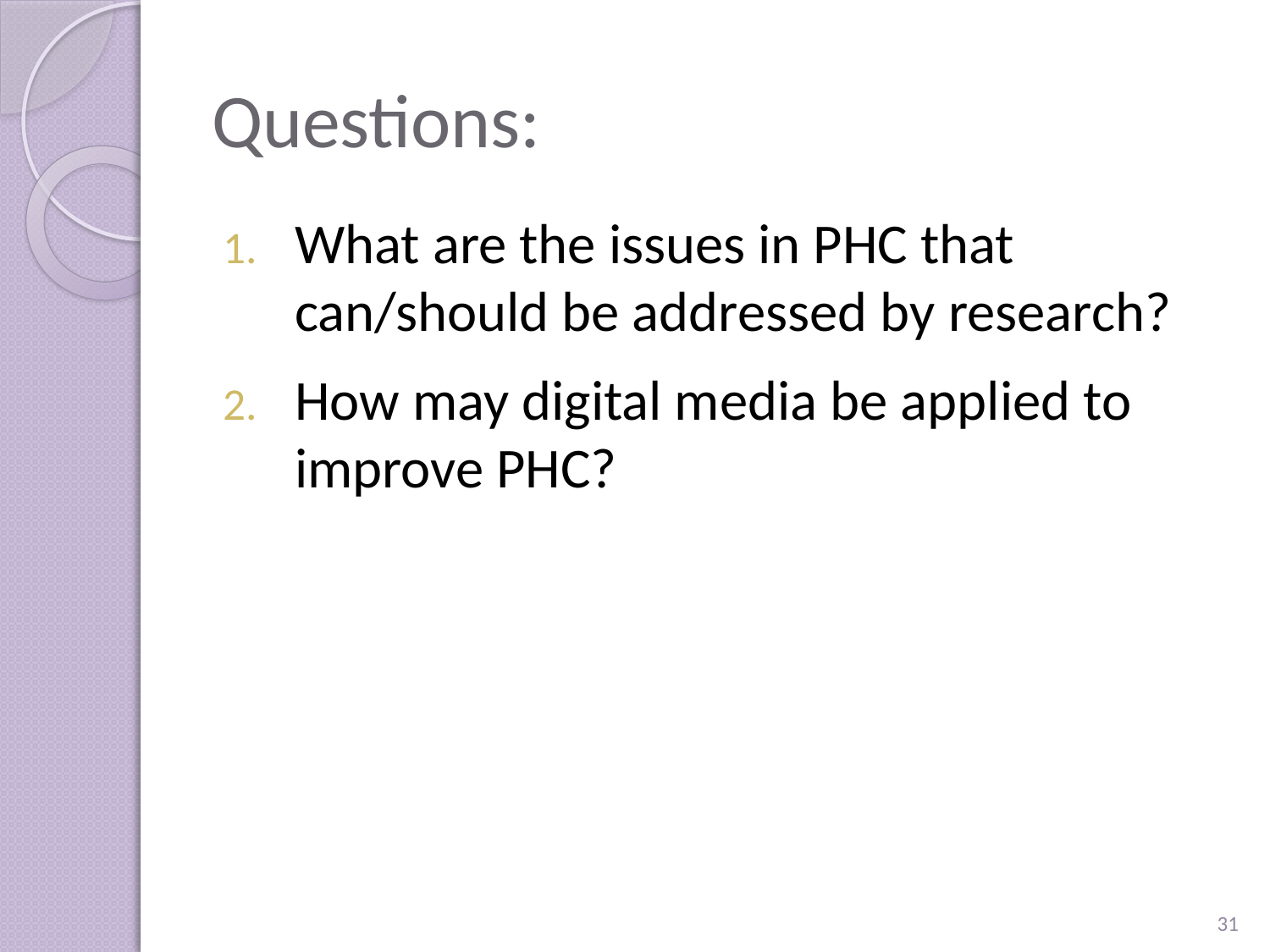

# Questions:
What are the issues in PHC that can/should be addressed by research?
How may digital media be applied to improve PHC?
31

## Slide 32
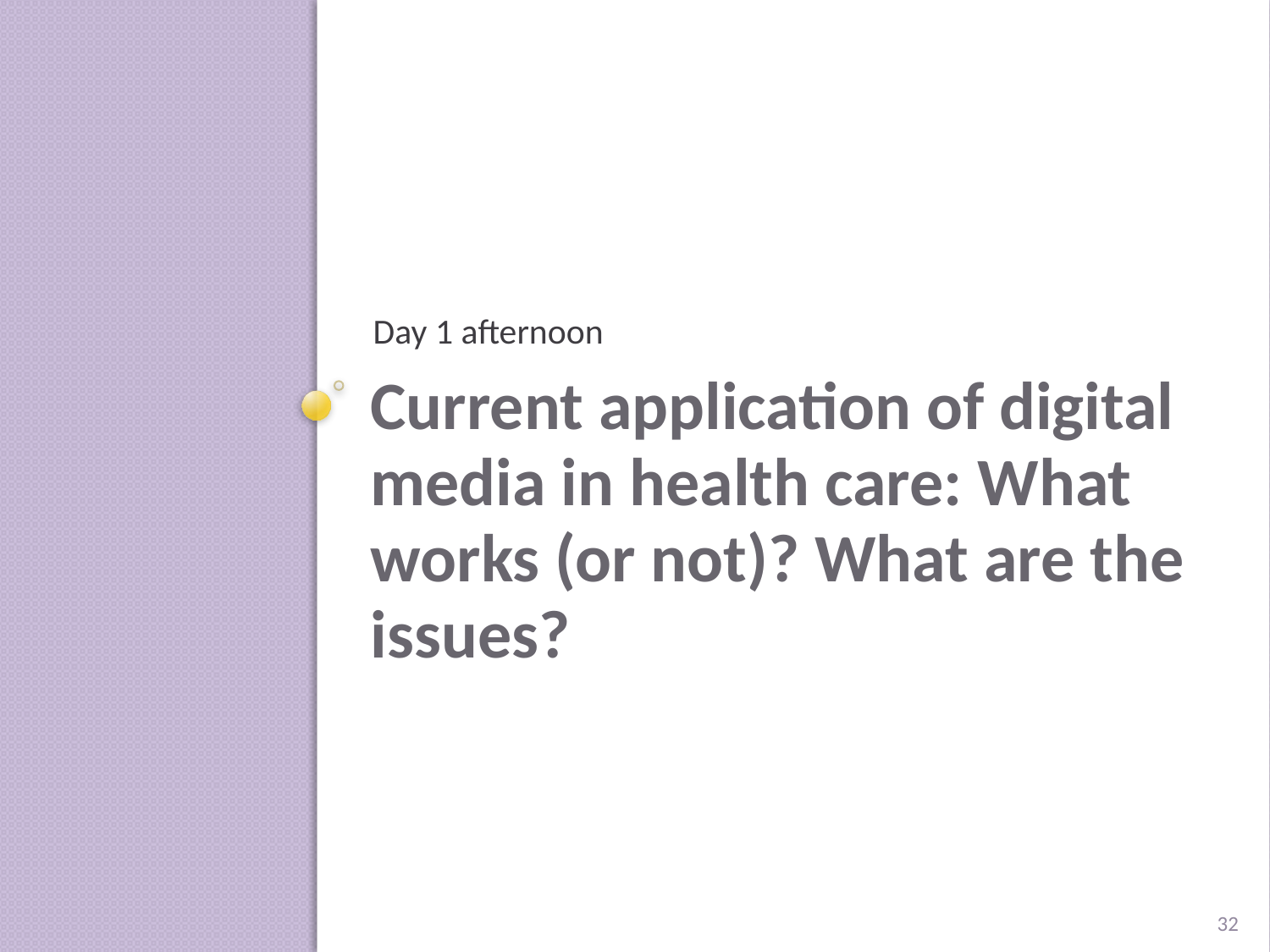

Day 1 afternoon
# Current application of digital media in health care: What works (or not)? What are the issues?
32

## Slide 33
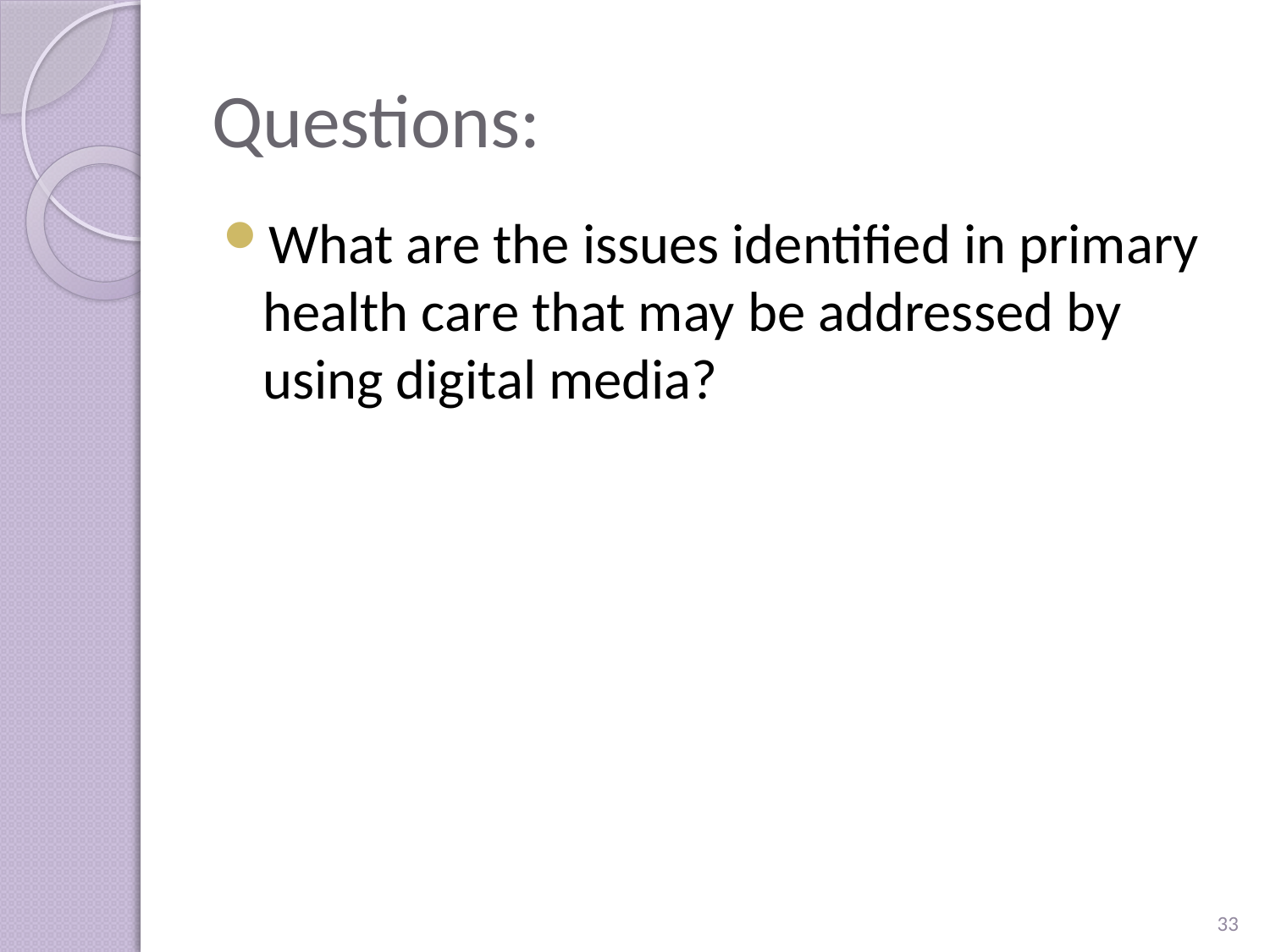

# Questions:
What are the issues identified in primary health care that may be addressed by using digital media?
33

## Slide 34
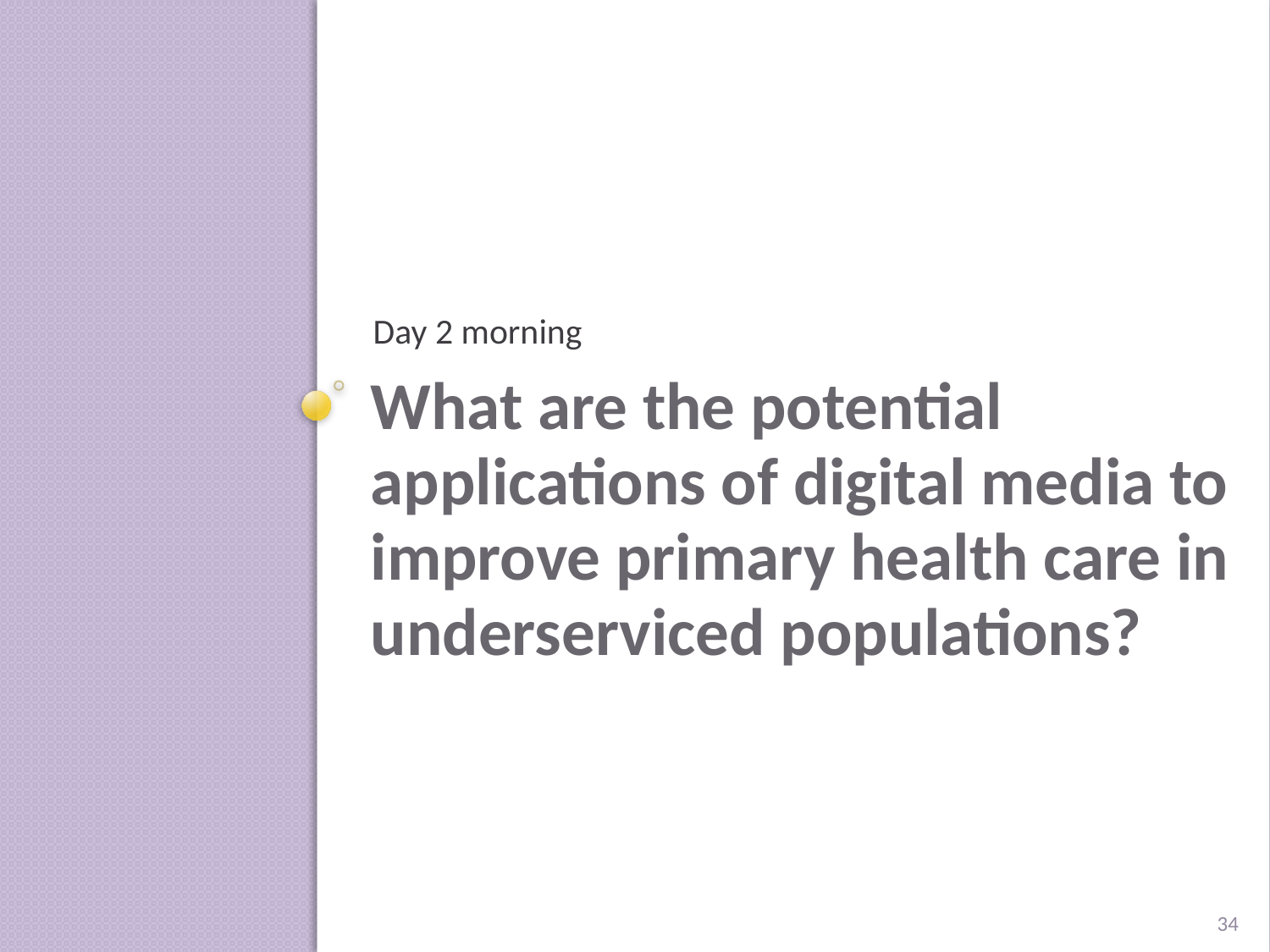

Day 2 morning
# What are the potential applications of digital media to improve primary health care in underserviced populations?
34

## Slide 35
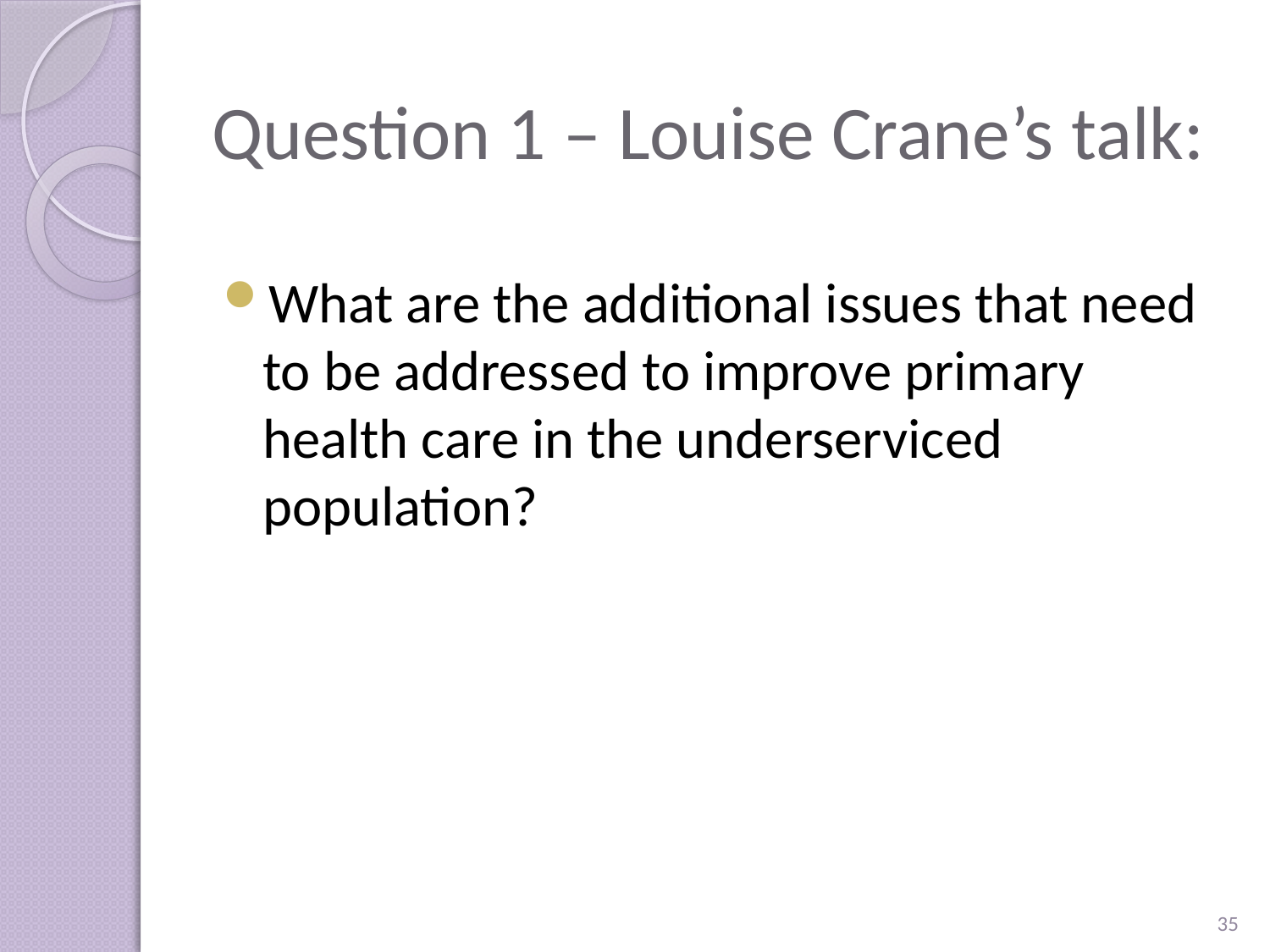

# Question 1 – Louise Crane’s talk:
What are the additional issues that need to be addressed to improve primary health care in the underserviced population?
35

## Slide 36
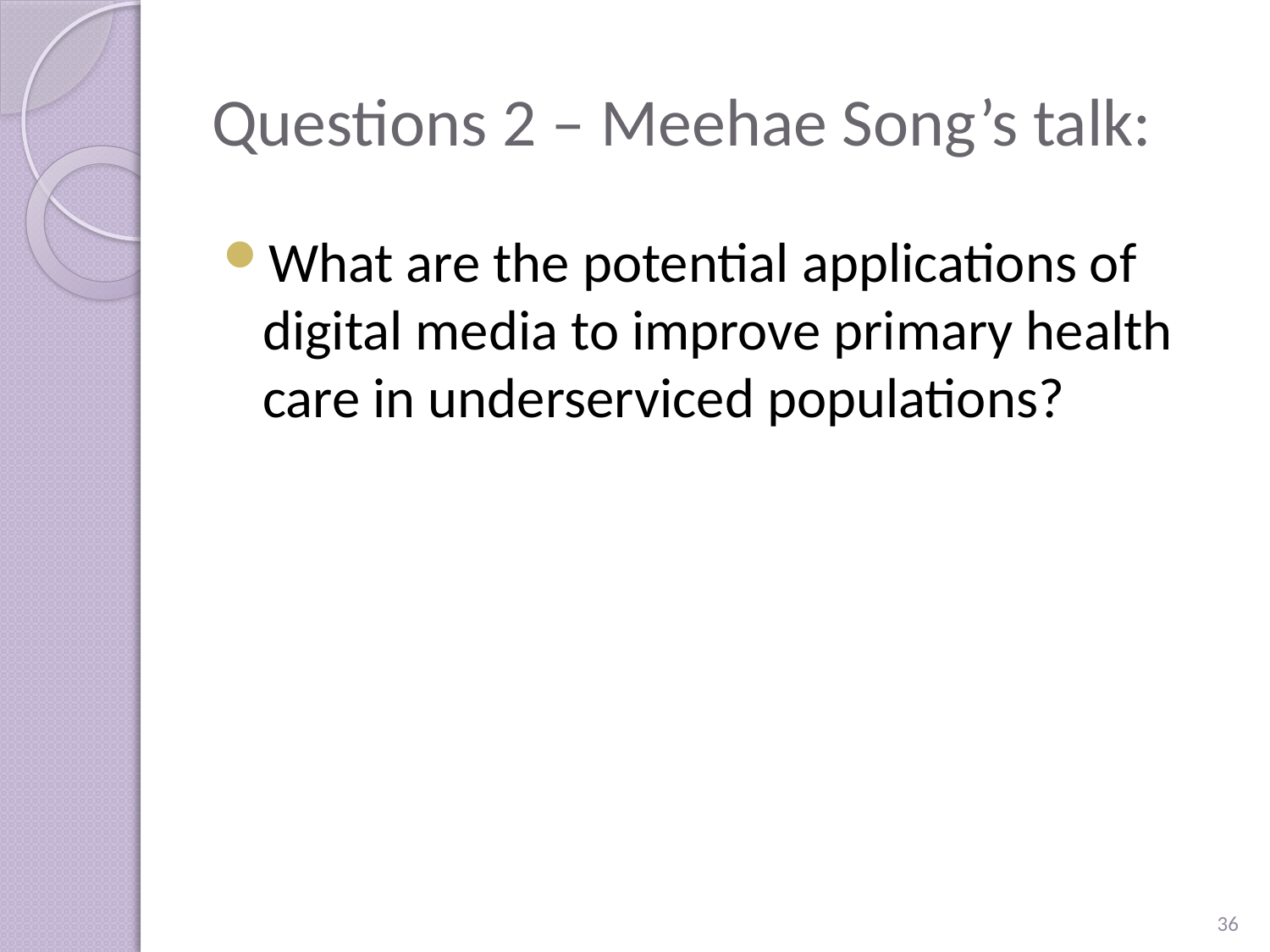

# Questions 2 – Meehae Song’s talk:
What are the potential applications of digital media to improve primary health care in underserviced populations?
36

## Slide 37
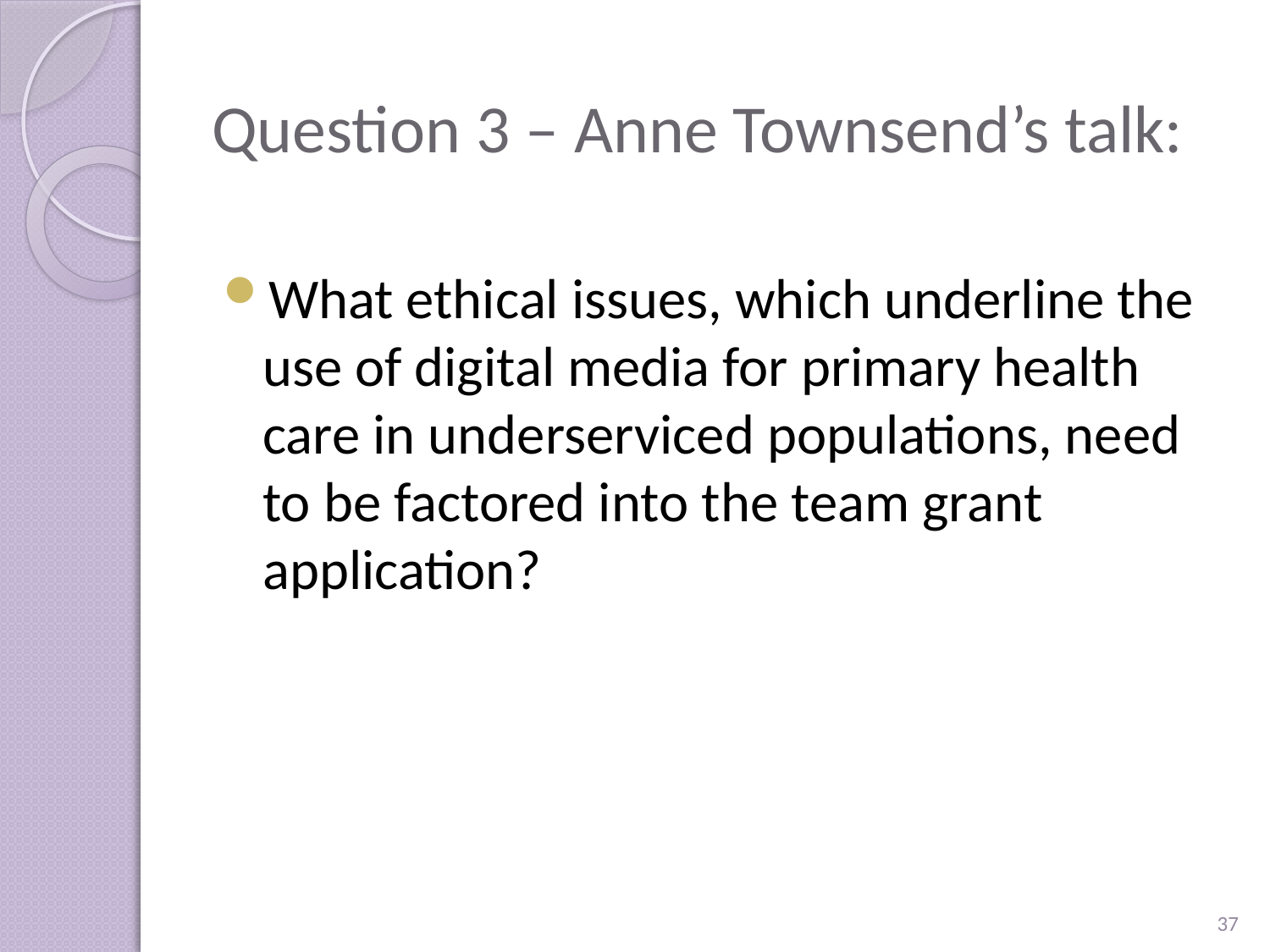

# Question 3 – Anne Townsend’s talk:
What ethical issues, which underline the use of digital media for primary health care in underserviced populations, need to be factored into the team grant application?
37

## Slide 38
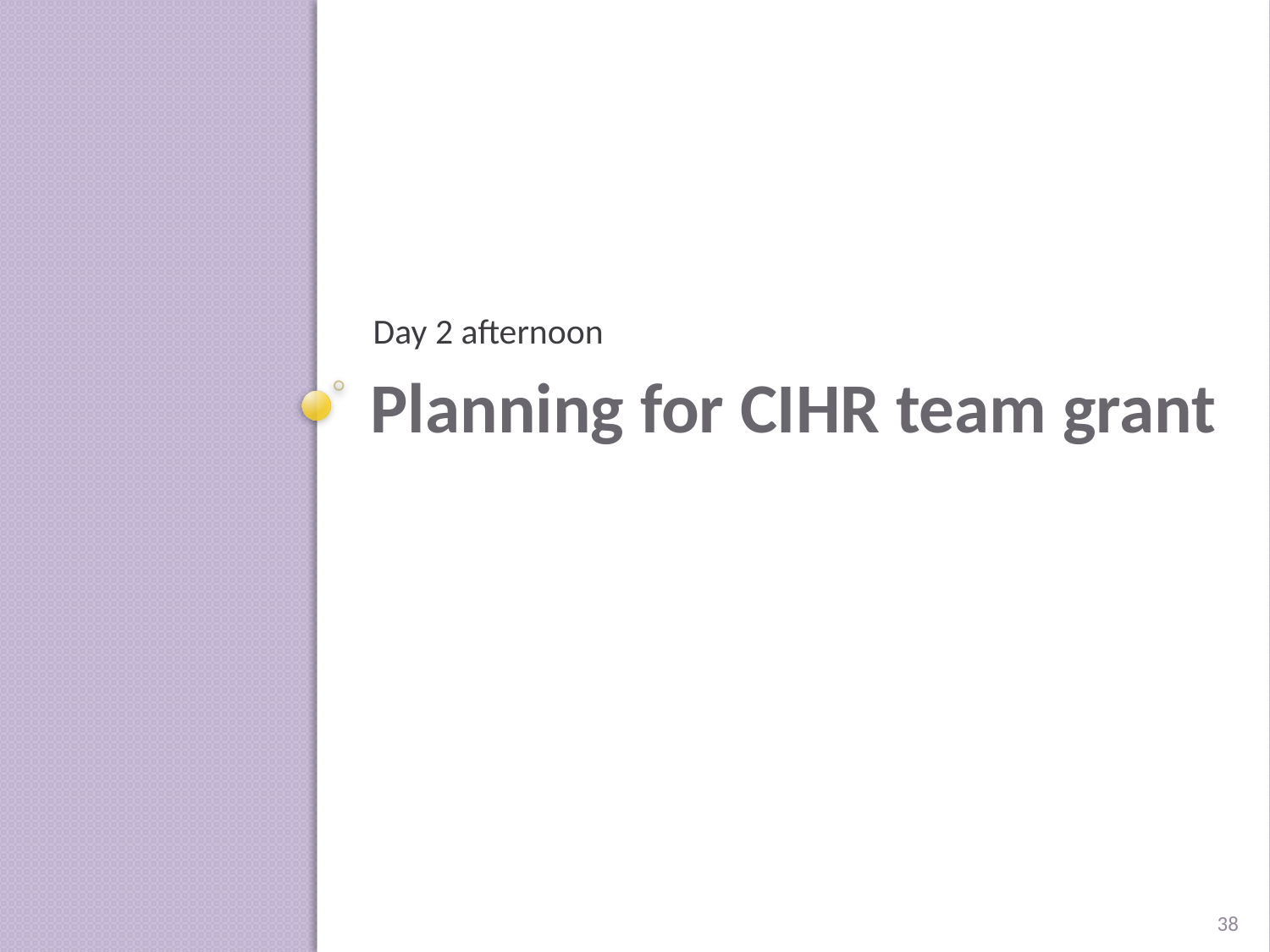

Day 2 afternoon
# Planning for CIHR team grant
38
